# Hydrogel Films in Biomedical Applications: Fabrication, Properties and Therapeutic Potential

**DOI:** 10.3390/gels11110918

**Published:** 2025-11-17

**Authors:** Sabuj Chandra Sutradhar, Hyoseop Shin, Whangi Kim, Hohyoun Jang

**Affiliations:** Department of Energy Materials Science and Engineering, Konkuk University, 268 Chungwon-daero, Chungju-si 27478, Republic of Korea; chandra@kku.ac.kr (S.C.S.); hyoseop.shin@toh.net.cn (H.S.); wgkim@kku.ac.kr (W.K.)

**Keywords:** hydrogel films, biomedical applications, stimuli-responsive materials, drug delivery systems, tissue engineering, implantable biosensors

## Abstract

Hydrogel films have emerged as versatile platforms in biomedical engineering due to their unique physicochemical properties, biocompatibility, and adaptability to diverse therapeutic needs. This review provides a comprehensive overview of hydrogel film materials, including natural biopolymers, synthetic polymers, and multifunctional composites, highlighting their structural and functional diversity. We examine key fabrication techniques—ranging from solvent casting and photopolymerization to advanced methods like microfluidics and 3D printing—and discuss how these influence film architecture and performance. The biomedical applications of hydrogel films span wound healing, drug delivery, tissue engineering, ophthalmology, and implantable biosensors, with recent innovations enabling stimuli-responsive behavior, multi-drug loading, and integration with wearable electronics. Despite their promise, hydrogel films face persistent challenges in mechanical durability, sterilization, storage stability, regulatory approval, and scalable manufacturing. We conclude by identifying critical research gaps and outlining future directions, including AI-guided design, sustainable material development, and the establishment of standardized, regulatory-aligned, and industrially scalable fabrication strategies to accelerate clinical translation.

## 1. Introduction

Hydrogels are three-dimensional networks of hydrophilic polymers capable of absorbing and retaining large amounts of water, making them one of the most versatile classes of biomaterials in biomedical science [1]. Their high-water content, softness, and biocompatibility allow them to closely mimic the extracellular matrix (ECM) of biological tissues such as skin, cartilage, and mucosa, which has led to their widespread use in wound healing, drug delivery, tissue engineering, and biosensing applications [2]. Traditionally, bulk hydrogels have dominated the field due to their excellent swelling behavior, permeability, and responsiveness to environmental stimuli such as pH, temperature, and ionic strength [3]. However, recent advances have shifted attention toward hydrogel films—thin, flexible layers of hydrogel materials with thicknesses ranging from nanometers to micrometers—which offer unique advantages in biomedical contexts [4].

Hydrogel films represent a significant evolution in hydrogel technology [5]. Their thin-film architecture allows for intimate contact with biological surfaces, enabling applications such as transdermal patches, implant coatings, ophthalmic devices, and bioelectronic interfaces [6,7,8,9,10]. Compared to bulk hydrogels, hydrogel films exhibit faster response times, greater surface adaptability, and enhanced integration with biomedical devices. These properties are particularly valuable in dynamic physiological environments where responsiveness and conformability are critical [11].

The fabrication of hydrogel films has advanced significantly, with techniques such as solvent casting [12], spin coating [13], layer-by-layer assembly [14], photopolymerization [15], and 3D printing [16], enabling precise control over film thickness, architecture, and functionality [17]. These methods allow for the incorporation of stimuli-responsive elements, bioactive molecules, and nanomaterials, transforming hydrogel films into smart systems capable of dynamic interaction with biological environments [18]. For example, thermo-responsive hydrogels like poly(N-isopropylacrylamide) (PNIPAm) exhibit phase transitions near body temperature, enabling on-demand drug release and real-time biosensing [19].

Hydrogel films can be composed of natural polymers such as chitosan, gelatin, and alginate, which offer excellent biocompatibility and biodegradability [20,21,22], or synthetic polymers like polyvinyl alcohol (PVA), polyacrylamide (PAAm), and polyethylene glycol (PEG), which provide tunable mechanical strength and chemical stability [23,24,25]. Hybrid hydrogels that combine natural and synthetic components are increasingly used to balance bioactivity with durability [26,27]. Crosslinking mechanisms—whether physical, chemical, or enzymatic—play a crucial role in determining the mechanical and biological properties of hydrogel films. Physically crosslinked hydrogels formed via hydrogen bonding or ionic interactions are reversible and suitable for dynamic applications, while chemically crosslinked hydrogels offer permanent structures ideal for long-term implantation [28,29,30].

In biomedical applications, hydrogel films have demonstrated exceptional promise [31]. In wound healing, they maintain a moist environment, absorb exudates, and deliver therapeutic agents, thereby accelerating tissue regeneration [32,33,34,35]. Self-healing hydrogel films that withstand mechanical stress during dressing changes further improve patient comfort and reduce secondary damage [36,37,38,39,40].

In drug delivery, hydrogel films serve as platforms for localized and sustained release, reducing systemic toxicity and improving therapeutic efficacy [41,42,43,44]. Their porous structure allows for high drug loading and controlled release kinetics, which can be tailored through polymer composition and crosslinking density. Stimuli-responsive hydrogel films can release drugs in response to environmental changes, such as pH shifts or temperature variations, enabling precision therapy [45]. Commercial hydrogel products have already been developed for transdermal, ocular, and injectable drug delivery routes, demonstrating the clinical viability of hydrogel film technologies [46].

In tissue engineering, hydrogel films act as scaffolds that support cell growth and differentiation [47,48,49]. Their ability to mimic the mechanical and biochemical properties of native tissues makes them suitable for regenerating skin, cartilage, and bone [50,51,52]. Composite hydrogel films incorporating bioactive molecules like hydroxyapatite or collagen have shown improved osteogenic and angiogenic potential [53]. Advanced fabrication techniques, such as 3D bioprinting, enable the creation of hydrogel films with complex architectures and spatial control over cell distribution, paving the way for personalized tissue constructs [54].

Despite their promising applications, hydrogel films face several challenges. Mechanical fragility, limited scalability, and variable degradation rates can hinder clinical translation [55,56,57]. Ensuring long-term biocompatibility and avoiding immune responses remain critical concerns. To address these issues, researchers are exploring nanocomposite hydrogels, bioinspired designs, and hybrid crosslinking strategies that enhance mechanical strength and biological performance [58]. The integration of hydrogel films with electronic components for biosensing and soft robotics is another emerging frontier. These systems can monitor physiological signals, deliver drugs, and respond to stimuli in real time, offering new possibilities for wearable and implantable medical devices [59].

**Scheme 1:**
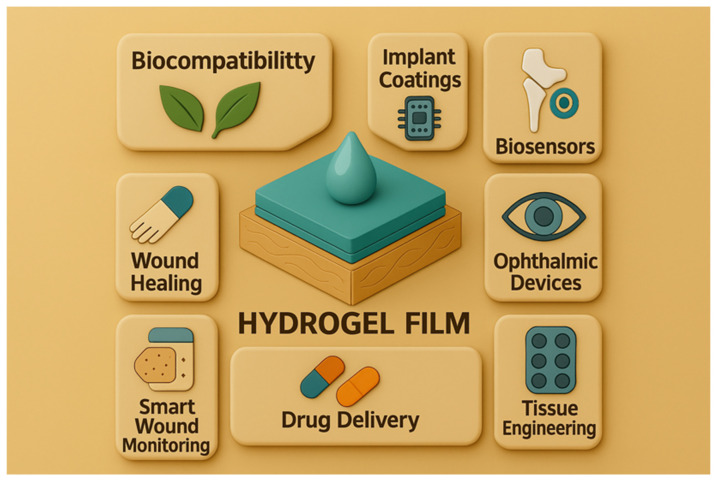
Schematic overview of the key properties and biomedical applications of hydrogel films.

In summary, hydrogel films represent a transformative advancement in biomaterials science, offering a unique combination of biocompatibility, functional versatility, and engineering precision (Scheme 1). Their ability to mimic biological tissues, respond to environmental cues, and deliver therapeutic agents positions them as key players in the future of personalized medicine, regenerative therapies, and bio-integrated technologies. As fabrication techniques and material designs continue to evolve, hydrogel films are poised to redefine the landscape of biomedical applications [60,61,62].

To support this perspective, the present review provides a structured and comprehensive overview of hydrogel films, beginning with their unique physicochemical properties and fabrication strategies. We then explore their diverse biomedical applications—including wound dressings, drug delivery systems, tissue engineering scaffolds, ophthalmic devices, and implantable biosensors—highlighting both established uses and emerging innovations. Recent advances in stimuli-responsive and nanocomposite hydrogel films are discussed, followed by an analysis of current challenges such as mechanical durability, sterilization, and scalability. Finally, we present future directions for hydrogel film technologies, including personalized designs, AI-guided optimization, and clinical translation, aiming to illuminate their growing impact in next-generation biomedical solutions.

## 2. Materials and Composition of Hydrogel Films

Hydrogel films are synthesized from a wide spectrum of polymers, each offering distinct physicochemical and biological properties. These materials are broadly categorized into natural biopolymers, synthetic polymers, and composite systems, often engineered for specific biomedical functionalities.

### 2.1. Natural Biopolymer-Based Hydrogel Films

Natural hydrogel films are increasingly recognized as sustainable and biocompatible platforms for biomedical applications. Derived from renewable biological sources such as algae, crustaceans, milk, plants, and insects, these materials offer a unique combination of biodegradability, bioactivity, and environmental compatibility. Their ability to absorb large quantities of water, release drugs in a controlled manner, and mimic extracellular matrices makes them ideal for applications in wound healing, drug delivery, and tissue engineering. However, challenges such as low mechanical strength, batch variability, and limited industrial scalability remain [63].

#### 2.1.1. Alginate

Alginate is a naturally occurring polysaccharide extracted from brown algae and certain bacteria [64]. It consists of alternating blocks of α-L-guluronic acid (G) and β-D-mannuronic acid (M) linked via 1,4-glycosidic bonds [65]. Sodium alginate is water-soluble but forms hydrogels upon ionic crosslinking with multivalent cations such as Ca^2+^, Mg^2+^, or Fe^3+^ [66]. The mechanical strength and water resistance of alginate films depend on the type and concentration of these crosslinkers. These films are flexible, transparent, and widely used in food packaging, cosmetics, and biomedical applications [67,68]. In biomedical contexts, alginate hydrogel films have shown particular promise in wound dressing and drug delivery. As reviewed by Abasalizadeh et al., these films maintain a moist environment, absorb wound exudates, and promote healing through autolytic debridement. Their hemostatic properties and ability to incorporate antimicrobial agents such as silver nanoparticles make them effective for managing chronic and acute wounds. In drug delivery, alginate films enable the controlled and localized release of therapeutic agents, with pH-sensitive and nanoparticle-integrated systems offering targeted delivery, especially in cancer treatment. Despite limitations in cell adhesion and mechanical strength, ongoing research into functionalization and composite formulations continues to expand their clinical potential [69].

#### 2.1.2. Chitosan

Chitosan is obtained by deacetylating chitin, a structural component found in the exoskeletons of crustaceans, insects, and fungi [70]. It is a linear copolymer of (1-4)-2-amino-2-deoxy-β-D-glucan (GlcN) and (1-4)-2-acetamido-2-deoxy-β-D-glucan (GlcNAc) [71]. Chitosan-based hydrogel films are flexible, transparent, and possess antimicrobial, antioxidant, and oxygen-barrier properties, making them suitable for wound dressings, oral drug delivery, and tissue scaffolds [72]. Chitosan can be chemically modified through grafting and derivatization, using its reactive amine and hydroxyl groups to attach functional side chains. These modifications—such as forming quaternary ammonium salts, esters, ethers, and Schiff bases—enhance its solubility, mechanical strength, and biocompatibility. The performance of chitosan hydrogels is influenced by molecular weight and source. For example, high-viscosity chitosan from sea urchin spiny powder yields hydrophobic and stable films [73], while medium-weight chitosan is preferred for nutraceuticals and cosmetic applications [74]. Chitosan hydrogel films have also demonstrated notable efficacy in biomedical applications. Ahmed et al. reported that chitosan-silver nanoparticle composite films exhibited enhanced antibacterial activity and accelerated wound healing in vivo, outperforming conventional dressings. In drug delivery, chitosan films enable localized and sustained release of therapeutics, with their mucoadhesive and pH-responsive properties supporting targeted delivery. Limitations such as poor solubility at physiological pH and low mechanical strength are being addressed through polymer blending and chemical modifications, reinforcing chitosan’s role as a versatile platform for advanced wound care and controlled drug release [75].

#### 2.1.3. Carrageenan

Carrageenan is a sulfated polysaccharide derived from red algae, composed of galactose and 3,6-anhydrogalactose units linked by alternating α-(1,3) and β-(1,4) glycosidic bonds. It exists in three main forms—kappa (κ), iota (ι), and lambda (λ)—based on sulfate content and gelation behavior [76]. Carrageenan hydrogels can be formed via physical or chemical crosslinking, and are often blended with other polymers such as agarose or chitosan to tailor mechanical and biological properties for drug delivery, biosensing, and tissue engineering [77]. Neamtu et al. (2022) [78] reviewed carrageenan-based compounds and highlighted their potential in wound healing applications, emphasizing their ability to form biocompatible hydrogels that support moisture retention, cellular regeneration, and controlled delivery of bioactive agents. The study also noted that kappa- and iota-carrageenan variants exhibit favorable gelation and mechanical properties, making them suitable for developing wound dressings and therapeutic platforms [78].

#### 2.1.4. Hyaluronic Acid

Hyaluronic acid (HA) is a non-sulfated glycosaminoglycan found in the extracellular matrix of connective tissues [79]. It consists of repeating disaccharide units of D-glucuronic acid and N-acetyl-D-glucosamine [80]. Crosslinking of HA chains can be achieved using vinyl ether–maleic anhydride copolymers [81]. HA-based films are known for their high water retention, swelling capacity, and tissue compatibility, making them suitable for cartilage repair, ocular applications, and injectable drug carriers. Recent studies have also demonstrated their efficacy in wound healing and drug delivery. For example, recent advances have focused on enhancing HA’s therapeutic functionality in wound healing. For example, the study by Zhao et al. (2025) [82] developed a multi-network hydrogel (GHrCT) incorporating dopamine-modified HA, tannic acid, and recombinant collagen type III. These hydrogels maintained a moist wound environment and supported sustained release of therapeutic agents, highlighting HA’s potential as a multifunctional biomaterial in regenerative medicine [82].

#### 2.1.5. Collagen

Collagen is a fibrous protein found in skin, bone, and cartilage, characterized by a triple-helix structure formed by repeating (Gly-X-Y)n sequences, where X and Y are typically proline and hydroxyproline [83,84]. Hydrogels are formed by crosslinking collagen chains using heat, UV light, or chemical agents like glutaraldehyde and carbodiimides [85]. These films mimic the extracellular matrix, supporting cell adhesion, proliferation, and tissue regeneration [86,87]. A recent study by Markandeywar et al. introduced a biogenic sprayable hydrogel composed of collagen, chitosan, and silver nanoparticles for advanced wound care. The formulation demonstrated excellent antibacterial activity, biocompatibility, and wound healing efficacy. In vitro and in vivo evaluations confirmed its ability to accelerate tissue regeneration, reduce inflammation, and prevent microbial infection, making it a promising candidate for next-generation wound dressings [88].

#### 2.1.6. Silk Fibroin

Silk fibroin is a natural protein produced by Bombyx mori and other insects. It consists mainly of glycine, alanine, and serine, arranged in repetitive sequences that form β-sheet crystalline domains [89,90,91]. Gelation can occur spontaneously via pH, temperature, or ultrasound, but is typically controlled through chemical or enzymatic crosslinking. Enzymes like HRP, glutaminase, and tyrosinase, or agents like genipin, glutaraldehyde, epoxides, and carbodiimides, react with active groups on fibroin chains to form stable networks [80,92]. Recent advances have demonstrated the versatility of silk fibroin in hydrogel film applications for wound healing and drug delivery. For example, Sheybanikashani et al. developed a sustainable, self-healing silk fibroin nanocomposite hydrogel incorporating antibacterial agents and therapeutic drugs for 3D-printed wound dressings. The hydrogel exhibited excellent elasticity, ROS-scavenging capacity, and sustained drug release, while in vivo studies confirmed enhanced wound closure, reduced infection, and improved tissue regeneration. This highlights silk fibroin’s potential as a multifunctional scaffold for advanced biomedical applications [93].

#### 2.1.7. Casein

Casein is the major protein in milk, forming micellar aggregates that can be denatured to produce hydrogels [94,95]. Crosslinking can be achieved physically (temperature), chemically (pH, ionic strength), or enzymatically (e.g., transglutaminase) [95]. The resulting networks may involve disulfide bridges, oligomer reactions, or crystallization zones [81]. Casein hydrogels are used in oral drug delivery, nutraceuticals, and bioadhesive films. In a study by Garcia et al., antiseptic-loaded casein hydrogels were developed and evaluated for wound healing efficacy [96]. The hydrogels demonstrated sustained release of antiseptic agents, maintained antimicrobial activity against common pathogens, and supported tissue regeneration in vitro. Their physicochemical properties—including swelling behavior, mechanical strength, and degradation rate—were optimized for prolonged skin contact. This research highlights casein’s potential as a natural, cost-effective platform for multifunctional wound care and localized drug delivery.

#### 2.1.8. Cellulose

Cellulose is a natural polysaccharide composed of β-(1→4)-linked D-glucose units [97,98], widely sourced from plants and bacteria. Its hydrophilic nature, biodegradability, and mechanical strength make it an attractive material for biomedical applications. Cellulose-based hydrogels are typically formed through physical entanglement or chemical crosslinking using agents like epichlorohydrin, citric acid, or carbodiimides. Chemical crosslinking is required to form stable hydrogels, using agents like dialdehydes, polycarboxylic acids, and epichlorohydrin [99,100]. These hydrogels exhibit excellent water retention, oxygen permeability, and compatibility with skin tissue, making them suitable for wound dressings, biosensors, and drug delivery systems. In a comprehensive review, Alven and Aderibigbe et al. highlighted the potential of cellulose-based hydrogels in wound management [101]. They discussed various formulations, including bacterial cellulose and cellulose derivatives (e.g., carboxymethyl cellulose), which demonstrated enhanced healing through moisture retention, antimicrobial activity, and controlled drug release. The review emphasized that cellulose hydrogels can be tailored to deliver antibiotics, anti-inflammatory agents, or growth factors, supporting tissue regeneration and reducing infection risk.

#### 2.1.9. Lignin

Lignin is a highly branched, amorphous biopolymer composed of phenylpropanoid units such as p-coumaryl, coniferyl, and sinapyl alcohols [102,103,104]. Its phenolic hydroxyl groups enable crosslinking via hydroxymethylation and epoxidation, using agents like formaldehyde and epichlorohydrin [105]. Despite its potential, lignin hydrogels face challenges due to high production costs, structural variability, and limited mechanical performance. In a 2024 study, Preet et al. developed a lignin-based biocomposite hydrogel for wound healing applications [106]. The hydrogel combined lignin with gelatin and silver nanoparticles, forming a matrix with excellent swelling capacity, porosity, and antibacterial performance. In vitro tests showed strong inhibition of microbial growth, while in vivo experiments on full-thickness wounds in rats revealed accelerated epithelialization, collagen deposition, and reduced inflammation. The hydrogel’s antioxidant activity also helped mitigate oxidative stress in the wound microenvironment, promoting faster and cleaner healing. This research highlights lignin’s potential as a bioactive component in hydrogel films, especially for multifunctional wound dressings that combine healing, protection, and drug delivery.

Natural polymers are favored for their biocompatibility, biodegradability, and bioactivity, making them ideal for applications involving direct tissue contact. However, their mechanical limitations often necessitate crosslinking or blending.

### 2.2. Synthetic Polymer-Based Hydrogel Films

Synthetic polymer hydrogels are engineered materials derived from man-made polymers, offering precise control over physicochemical properties, scalability, and customizability for diverse biomedical applications [107]. Unlike natural hydrogels, synthetic variants can be tailored to exhibit specific mechanical strength, degradation rates, and stimuli responsiveness, making them ideal for drug delivery, tissue scaffolding, biosensing, and wound healing [108].

The most commonly used synthetic polymers in hydrogel film fabrication include polyvinyl alcohol (PVA), polyethylene glycol (PEG), poly(hydroxyethyl methacrylate) (pHEMA), poly(acrylic acid) (PAA), polyacrylamide (PAM), and poly(lactic-co-glycolic acid) (PLGA).

#### 2.2.1. Polyvinyl Alcohol (PVA)

PVA is a water-soluble synthetic polymer with the idealized formula [CH_2_CH(OH)]_n_. Hydrogels based on PVA can be formed via physical crosslinking, such as hot-pressing, which avoids organic solvents and yields transparent films, or via chemical crosslinking using agents like glutaraldehyde, which forms strong covalent bonds between polymer chains [109]. PVA hydrogels are widely used in ocular applications, wound dressings, and drug delivery systems due to their biocompatibility and film-forming ability. A systematic review by Annisa et al. (2023) emphasized the development of film-forming PVA hydrogels for wound dressing applications [110]. The study explored various formulations incorporating bioactive compounds, such as antiseptics and natural extracts, which enhanced antimicrobial activity and supported tissue regeneration. Additionally, Oliveira et al. (2024) investigated PVA and PVA–PVA-carboxymethyl cellulose (CMC) composite hydrogels loaded with natural extracts [111]. Their research demonstrated sustained drug release governed by power-law swelling behavior, confirming the hydrogels’ potential for controlled delivery and prolonged therapeutic action.

A broader review by Khan and Rumon (2023) outlined recent trends in PVA-based hydrogels for biomedical applications, including wound healing, tissue engineering, and drug delivery [109]. They highlighted PVA’s adaptability when combined with other polymers or nanoparticles to improve bioactivity and mechanical resilience.

#### 2.2.2. Polyethylene Glycol (PEG)

PEG, also known as polyethylene oxide (PEO) or polyoxyethylene (POE), is synthesized via the polymerization of ethylene oxide [112]. It is a non-toxic, water-soluble, and biocompatible polymer. PEG hydrogels are typically formed by chemical modification, such as acylation with acryloyl chloride to produce PEG diacrylate (PEG-DA). These derivatives can be crosslinked via UV light, enzymatic reactions, or chemical agents like diisocyanates and triols [113]. PEG-based hydrogels are used in injectable systems, biosensors, and controlled drug release platforms [114]. In a 2022 study, Afrin et al. developed a semi-interpenetrating polymer network (semi-IPN) hydrogel composed of cellulose nanocrystals (CNC), PEG, and poly(dimethylacrylamide) (PDMAA) [115]. This CNC/PEG/PDMAA hydrogel demonstrated excellent swelling behavior, mechanical strength, and biocompatibility. It was designed to manage drug delivery in wound healing applications, showing controlled release of therapeutic agents and enhanced healing efficacy. The incorporation of CNC improved the structural integrity and responsiveness of the hydrogel, while PEG contributed to its hydrophilicity and flexibility. This research underscores PEG’s versatility in forming multifunctional hydrogel films that can be tailored for various biomedical needs, including localized drug delivery and regenerative therapies.

#### 2.2.3. Poly(hydroxyethyl methacrylate) (pHEMA)

pHEMA is synthesized from hydroxyethyl methacrylate (HEMA) monomers, with the formula H_2_C=C(CH_3_)CO_2_CH_2_CH_2_OH [116]. It contains hydrophilic hydroxyethyl side groups, which facilitate water absorption. Hydrogel films made from pHEMA are typically synthesized via free-radical polymerization, often using crosslinking agents like ethylene glycol dimethacrylate (EGDMA) or triethylene glycol dimethacrylate (TEGDMA) [117]. pHEMA hydrogels are known for their optical clarity, softness, and biocompatibility, making them suitable for contact lenses, ocular implants, and tissue scaffolds. These films exhibit excellent swelling behavior, mechanical stability, and permeability, which are critical for wound healing and drug delivery. Their porous structure allows for the controlled release of therapeutic agents while maintaining a moist environment conducive to tissue regeneration. A comprehensive review by Zare et al. (2021) highlighted the versatility of pHEMA in biomedical applications, including its use in tissue engineering scaffolds, biosensors, and drug-eluting systems [116]. The authors emphasized that pHEMA hydrogels can be functionalized with bioactive molecules or blended with other polymers (e.g., PEG, chitosan) to enhance cell adhesion, antimicrobial activity, and mechanical performance.

#### 2.2.4. Poly(acrylic acid) (PAA)

PAA is formed by polymerizing acrylic acid, which contains carboxylic acid groups (-COOH) on each monomer unit [118]. These groups confer anionic character and high hydrophilicity, enabling exceptional water absorption. Crosslinking is commonly achieved via ionizing radiation, such as electron beams or gamma rays [119]. PAA hydrogels are used in wound dressings due to their adhesive properties [120] and in environmental sensors for detecting heavy metal ions in aqueous solutions [121]. Recent studies have expanded their biomedical relevance. For instance, Dong et al. (2024) developed a multifunctional hydrogel adhesive composed of protocatechuic acid and PAA, which exhibited strong tissue adhesion, antioxidant activity, and antibacterial properties [122]. The hydrogel promoted wound healing by maintaining a moist environment, reducing oxidative stress, and preventing microbial infection. Similarly, Oouchi et al. (2024) reported on bioadhesive PAA/polyvinylpyrrolidone (PVP) complex gels that enhanced wound healing through improved elasticity, moisture retention, and prolonged adherence to wound surfaces [123]. These findings underscore the versatility of PAA-based hydrogels as multifunctional platforms for advanced wound care.

#### 2.2.5. Polyacrylamide (PAM)

PAM, with the formula (-CH_2_CHCONH_2_-)_n_, is a linear-chain polymer known for its water retention and ionic strength buffering capabilities [124]. PAM hydrogels are synthesized via the redox polymerization of acrylamide monomers and crosslinked using N, N′-methylene bis-acrylamide. The amide functional groups facilitate water absorption and interaction with embedded molecules. PAM hydrogels are applied in ophthalmic surgery, drug delivery, food packaging, and water purification. Recent studies have expanded their role in wound healing and transdermal therapy. For instance, Li et al. (2024) developed a PAM-based hydrogel incorporating platelet-rich plasma (PRP) for wound healing, activated by low-intensity ultrasound to enable sustained release and enhanced tissue regeneration [125]. Similarly, Kim et al. (2024) engineered a PAM/polydopamine adhesive hydrogel patch for transdermal vitamin E delivery, demonstrating strong adhesion, antioxidant activity, and controlled drug release [126]. Jiang et al. (2023) introduced a temperature-responsive PAM hydrogel dressing capable of real-time and remote wound monitoring, showcasing PAM’s adaptability in smart biomedical systems [127].

#### 2.2.6. Poly(N-isopropylacrylamide) (PNIPAM)

Poly(N-isopropylacrylamide) (PNIPAM) is a synthetic polymer widely studied for its temperature-responsive behavior, undergoing a reversible phase transition near 32 °C, which is close to human body temperature. This thermoresponsive property makes PNIPAM-based hydrogel films particularly attractive for biomedical applications that require dynamic responsiveness, such as drug delivery, wound dressing, tissue engineering, and biosensing. According to Narayana et al. (2025) [128], PNIPAM hydrogels have been effectively utilized in systems that release drugs in response to elevated temperatures, such as during inflammation or fever, ensuring targeted and timely therapeutic action. In wound care, PNIPAM-based films can regulate hydration and permeability based on skin temperature, maintaining an optimal healing environment. In tissue engineering, their phase transition behavior supports cell adhesion and proliferation, making them suitable for 3D scaffolds. Additionally, their integration into wearable biosensors enables real-time physiological monitoring through temperature-triggered signal modulation. The review also emphasizes the potential of enhancing PNIPAM’s performance through copolymerization with hydrophilic or biodegradable polymers and the incorporation of nanomaterials to improve mechanical strength, biocompatibility, and responsiveness. These modifications expand the utility of PNIPAM hydrogels across a wide range of biomedical platforms, positioning them as foundational materials in the development of next-generation smart hydrogel films.

#### 2.2.7. Poly(lactic-co-glycolic acid) (PLGA)

PLGA is a biodegradable copolymer synthesized via ring-opening polymerization of glycolide and lactide, the cyclic dimers of glycolic acid and lactic acid, respectively [129]. The polymer can be tailored by adjusting the molar ratio of lactide to glycolide (e.g., PLGA 75:25), which influences its crystallinity, degradation rate, and mechanical properties. PLGA hydrogels are extensively used in therapeutic devices, drug delivery systems, and tissue engineering scaffolds due to their biocompatibility and controlled degradation. Recent developments have demonstrated PLGA’s versatility in hydrogel formulations. Visan and Negut (2024) reviewed PLGA hydrogels designed for the sustained delivery of therapeutic agents, emphasizing their tunable degradation profiles and compatibility with both hydrophilic and hydrophobic drugs [130]. These hydrogels have been applied in localized cancer therapy, wound healing, and regenerative medicine. Additionally, PLGA nanoparticles have been explored for transdermal drug delivery, offering controlled release and enhanced skin penetration. Such innovations underscore PLGA’s adaptability in forming multifunctional hydrogel systems for advanced biomedical applications [131].

Hydrogels derived from synthetic polymers offer repeatable, scalable, and customizable solutions for biomedical engineering. Their industrial viability, mechanical robustness, and stimuli-responsive behavior make them superior in applications requiring precision and consistency. However, care must be taken to remove residual monomers, catalysts, and toxic reagents from the synthesis process to ensure biocompatibility, especially for clinical use. Synthetic polymers offer tunable mechanical strength, controlled degradation, and stimuli responsiveness. They are often blended with natural polymers to balance biofunctionality and durability.

### 2.3. Additives for Hydrogel Films

The performance and functionality of hydrogel films are not solely determined by their base polymer composition or crosslinking density. A critical dimension of hydrogel engineering involves the incorporation of additives, which can significantly enhance or tailor properties such as mechanical strength, thermal stability, barrier performance, electrical conductivity, and stimuli responsiveness [132,133].

These additives span a wide range of chemical classes and morphologies, including:

- Inorganic nanoparticles (e.g., noble metals, oxides);
- Nanoclays;
- Carbon-based nanostructures (e.g., graphene, graphene oxide);
- Organic nanostructures (e.g., liposomes);
- Metal–organic frameworks (MOFs).

#### 2.3.1. Inorganic Nanoparticles (e.g., Noble Metals, Oxides)

Additives can be introduced into hydrogel matrices via:

- In situ synthesis: Nanoparticles are generated directly within the hydrogel network during gelation.
- Ex situ blending: Pre-synthesized nanoparticles are physically or chemically embedded into the hydrogel.

For hydrogel films, innovative incorporation methods are often required to ensure uniform dispersion and stable integration. For example, silver nanoparticles have been synthesized in situ within calcium alginate films using spin coating techniques, enabling antibacterial functionality without compromising film integrity [134].

Novel metal nanoparticles such as silver (Ag) and gold (Au) are among the most widely used additives in hydrogel systems due to their antimicrobial activity, optical properties, and ease of synthesis. These particles can be formed via controlled chemical reduction within the hydrogel matrix [135,136]. Their inclusion enhances antibacterial performance, making them ideal for wound dressings, biosensors, and antimicrobial coatings. For example, Markandeywar & Narang developed a sprayable hydrogel composed of collagen, chitosan, and silver nanoparticles [88]. The hydrogel exhibited potent antibacterial activity and accelerated wound healing, making it suitable for advanced wound care applications.

Oxide nanoparticles such as ZnO, CuO, and TiO_2_ offer antibacterial, photocatalytic, and adsorptive properties. They can be engineered to selectively bind or degrade specific molecules, making them useful for drug delivery, environmental remediation, and bioactive films [29]. Iron oxide nanoparticles, in particular, introduce magnetic responsiveness, allowing for the remote control of hydrogel behavior (e.g., swelling, drug release) via external magnetic fields [137]. Moreover, many metal and oxide nanoparticles contribute to crosslinking, either physically (via ionic interactions) or chemically (via covalent bonding), thereby enhancing the structural integrity of the hydrogel matrix.

#### 2.3.2. Nanoclays

Nanoclays, such as montmorillonite, kaolinite, and halloysite, are layered silicate minerals that can be intercalated into hydrogel films to improve mechanical strength, barrier properties, and drug release control [138]. These hybrid systems are particularly promising for targeted drug delivery and food packaging, although further research is needed to optimize their dispersion and compatibility. A study by Gaharwar et al. (2014) incorporated laponite nanoclay into PEG-based hydrogels, resulting in enhanced mechanical integrity and sustained drug release for tissue engineering applications [139].

#### 2.3.3. Carbon-Based Nanostructures (e.g., Graphene, Graphene Oxide)

Graphene-based additives are incorporated into hydrogels via self-assembly or supramolecular interactions, forming hybrid films with enhanced mechanical, thermal, and electrical properties [140]. Graphene’s two-dimensional structure and high conductivity make it ideal for biosensing, electroactive scaffolds, and smart drug delivery systems. The performance of these films can be tuned by adjusting graphene concentration, polymer composition, and processing conditions. For example, Boobphahom et al. (2021) developed a TiO_2_/MXene-PVA/GO hydrogel-based electrochemical sensor for neurological disorder screening via urinary norepinephrine detection [141]. This study developed a composite hydrogel using polyvinyl alcohol (PVA) and graphene oxide (GO), enhanced with TiO_2_ and MXene nanoparticles, to create a flexible and conductive material for biosensing applications. The hydrogel was applied to a screen-printed carbon electrode for detecting norepinephrine in urine, demonstrating high sensitivity and mechanical robustness—qualities that make it suitable for wearable biosensors and smart wound dressing platforms.

#### 2.3.4. Liposomes and Organic Nanostructures

Liposomes and other organic nanostructures can be embedded into hydrogels to facilitate drug encapsulation, controlled release, and biocompatibility [142,143]. These systems are especially useful for transdermal delivery, gene therapy, and cosmetic formulations, where targeted release and biological interaction are critical. Ternullo et al. (2020) developed a novel wound dressing by embedding curcumin-loaded deformable liposomes into a chitosan hydrogel matrix, resulting in a biocompatible system that provided sustained anti-inflammatory effects and enhanced healing in burn wound models [144]. The liposomal encapsulation improved curcumin’s stability and skin penetration, while the chitosan hydrogel offered a moist environment conducive to tissue regeneration, making the composite formulation highly effective for topical therapeutic applications.

#### 2.3.5. Metal–Organic Frameworks (MOFs)

MOFs are crystalline materials composed of metal ions coordinated to organic ligands, forming porous structures. When integrated into hydrogels, MOFs offer high drug loading capacity, selective adsorption, and stimuli-responsive behavior [145,146]. MOF–hydrogel films are used in drug delivery, biosensing, and chemical detection, leveraging the selectivity of MOFs and the biocompatibility of hydrogels. For example, Behjat et al. (2025) developed a multifunctional hydrogel film by incorporating ZIF-8 metal–organic frameworks (MOFs) into a polyvinyl alcohol (PVA) matrix, enhanced with tannic acid [147]. The resulting PVA/ZIF-8@TA hydrogel exhibited pH-responsive behavior, targeted antimicrobial activity, and reduced cytotoxicity. Designed for sustained delivery of garlic extract, the hydrogel demonstrated self-healing properties and strong antibacterial performance, making it a promising candidate for advanced wound dressing and controlled drug release applications.

### 2.4. Synergistic Additive Systems

Combining multiple additives can yield synergistic effects, enhancing multiple properties simultaneously. In addition to chemical additives, hydrogel performance can be enhanced by blending two or more biopolymers to create composite matrices with synergistic properties. These mixed systems often combine the mechanical strength, bioadhesiveness, and biocompatibility of different polymers for targeted biomedical applications. For instance, chitosan films blended with cerium oxide and graphene oxide demonstrated high antioxidant activity, low water solubility, and reduced moisture transmission, making them suitable for food packaging and biomedical barriers [148]. Additionally, Wathoni et al. (2019) developed a chitosan–alginate hydrogel film loaded with α-mangostin for treating recurrent aphthous stomatitis [149]. The composite film exhibited improved mucoadhesion, controlled drug release, and anti-inflammatory effects. Stubbe et al. (2019) formulated gelatin–alginate hydrogels for burn wound treatment. The dual-polymer system provided enhanced structural integrity, moisture retention, and supported cell proliferation and healing [150].

In summary, the strategic use of additives transforms hydrogel films from passive carriers into multifunctional platforms capable of active sensing, targeted delivery, and environmental responsiveness. The choice of additive, incorporation method, and matrix compatibility must be carefully optimized to achieve the desired performance in biomedical contexts. Composite hydrogels integrate nanoparticles, liposomes, or metal–organic frameworks (MOFs) to enhance drug loading, mechanical strength, and responsiveness.

To provide a clearer understanding of the strategic selection of hydrogel film types in biomedical applications, Table 1 presents a comparative analysis of natural biopolymer-based, synthetic polymer-based, and additive-enhanced composite hydrogels. Each category offers distinct advantages and limitations in terms of biocompatibility, mechanical performance, functional versatility, and scalability. This comparison is essential for guiding material choice based on application-specific requirements such as drug delivery, tissue engineering, biosensing, or wound healing.

## 3. Synthesis and Fabrication Techniques of Hydrogel Films

### 3.1. Chemical Crosslinking and Physical Gelation Methods

The synthesis of hydrogel films often employs chemical crosslinking and physical gelation techniques to establish stable polymer networks. Ionic crosslinking is widely used, particularly with biopolymers such as alginate and chitosan, where divalent cations or polyelectrolyte complexes facilitate network formation. Additionally, UV-induced polymerization enables spatial and temporal control over crosslink density, allowing for the fabrication of films with tailored mechanical and swelling properties. Schiff base formation, a reaction between amino and aldehyde groups, has been utilized to create dynamic covalent crosslinks within chitosan–alginate networks, enhancing film stability and facilitating controlled degradation [149,153,154].

Crosslinking agents such as glutaraldehyde, potassium persulfate, and sodium tripolyphosphate play critical roles in modulating the properties of hydrogel films. Their concentration and reaction conditions influence gelation time, mechanical strength, and swelling capacity. For example, potassium persulfate initiates free radical polymerizations, yielding biodegradable films with desirable hardness and swelling in biopolymeric systems. Crosslink density often inversely affects swelling but positively impacts mechanical robustness, reflecting the trade-offs that must be balanced during fabrication [153,155,156].

### 3.2. Fabrication Methods of Hydrogel Films

The fabrication of hydrogel films is a critical determinant of their structural, mechanical, and functional properties. Various methods have been developed to tailor hydrogel films for specific biomedical applications, ranging from wound dressings and drug delivery systems to biosensors and tissue scaffolds. This section discusses both conventional and emerging fabrication techniques, supported by recent research.

#### 3.2.1. Film Formation

Hydrogel films can be fabricated using various techniques, which differ in their applicability depending on the material system and desired properties. These methods are generally categorized based on the timing of gelation relative to polymerization and film deposition. One key distinction is between in situ crosslinking and post-synthetic crosslinking. In the in situ approach, polymer chains are formed directly from monomers or oligomers during film deposition, with crosslinking occurring simultaneously to establish the hydrogel network [157]. This process can be initiated by chemical agents or physical stimuli such as UV radiation, plasma, or thermal energy, which promote bond formation and network stabilization [158].

**Figure 1:**
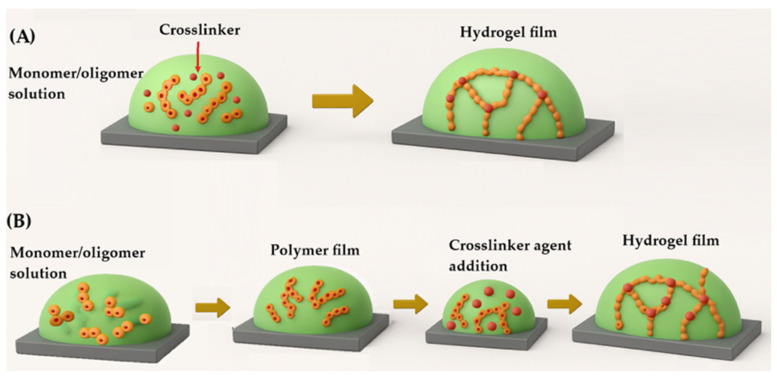
Schematic depiction of (**A**) in situ polymerization and gel formation through direct crosslinking, and (**B**) a post-synthetic crosslinking approach involving sequential steps: precursor solution deposition onto a substrate, polymer film formation, introduction of a crosslinking agent, and subsequent gelation.

In contrast, post-synthetic crosslinking involves first depositing a polymer film from a soluble precursor onto a substrate, followed by a separate crosslinking step. This secondary process may involve chemical crosslinkers or physical treatments like heat or irradiation, depending on the material and application [159]. Figure 1 presents a schematic representation outlining the two possible methods for film preparation.

Interestingly, even when strong covalent bonds are present within the hydrogel matrix, the adhesion between the hydrogel film and the substrate is often governed by van der Waals interactions. These weak forces between polymer chains and the substrate surface allow hydrogel coatings to adhere without requiring specific surface functionalization or pre-treatment, making them compatible with a wide range of materials [160].

Beyond conventional crosslinking methods, microwave-assisted synthesis has emerged as a promising alternative. This technique uses microwave irradiation to induce crosslinking in aqueous polymer solutions, offering advantages such as shorter reaction times, reduced chemical waste, and higher product yields [161,162]. For example, Sun et al. utilized this method to fabricate carbon dot-crosslinked sodium alginate hydrogel films, while Thongsuksaengcharoen et al. applied it to prepare PVA/PVP/CA hydrogel systems [161,163].

Microwave irradiation not only facilitates efficient crosslinking but also minimizes the need for chemical crosslinkers, potentially resulting in safer and cleaner hydrogel products. This makes it particularly attractive for biomedical applications where biocompatibility and purity are essential.

#### 3.2.2. Preparation Methods

**Figure 2:**
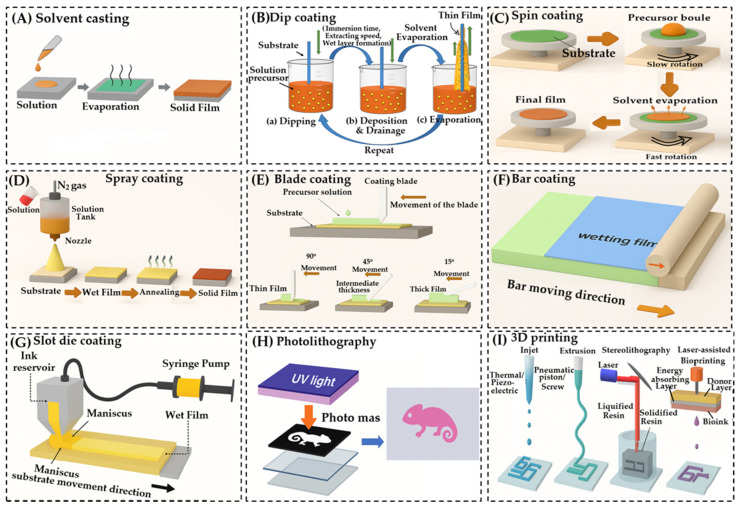
Schematic representation of hydrogel film fabrications. (**A**) The solvent casting method; (**B**) dip coating; (**C**) spin coating; (**D**) spray coating; (**E**) blade coating; (**F**) bar coating; (**G**) slot die coating; (**H**) photolithography method; (**I**) 3D printing techniques, respectively.

Hydrogel film fabrication has transitioned from basic casting techniques to sophisticated, precision-controlled processes. The choice of method depends on polymer chemistry, substrate interaction, desired film properties, and scalability. Figure 2 represents the schematic hydrogel film fabrication methods. Recent advances have focused on enhancing mechanical robustness, stimuli responsiveness, and integration with biomedical and electronic systems.

#### 3.2.3. Conventional Techniques and Their Evolution

- Solvent Casting remains a foundational method due to its simplicity and low cost. It involves dissolving polymers in a solvent, casting onto a substrate, and drying [164]. Recent studies have optimized this method by incorporating nanoparticles, liposomes, and metal–organic frameworks to improve biocompatibility and functionality [3]. However, limitations include residual solvent toxicity and lower mechanical strength.
- Dip Coating offers uniform coverage on complex geometries [165]. Advances include automated withdrawal systems and controlled drying environments, which improve reproducibility and film uniformity [166].
- Spin Coating is ideal for ultrathin films with high uniformity [13]. Recent developments have focused on rheological tuning of precursor solutions to achieve precise thickness control and rapid responsiveness, especially for biosensors and soft electronics [3].
- Spray, Slot Die, Blade, and Bar Coating are increasingly used in industrial settings for scalable production. Slot die coating, in particular, allows precise control over film thickness, making it suitable for membranes and flexible electronics [167,168,169,170].
- Photolithography enables microstructured hydrogel films with high spatial resolution. Innovations include UV-curable hybrid polymers and multi-layer patterning, useful for neural interfaces and microfluidic devices [171].

#### 3.2.4. Emerging Technologies

- 3D and 4D Printing: Additive manufacturing has revolutionized hydrogel fabrication. Techniques like extrusion-based printing, stereolithography (SLA), and digital light processing (DLP) allow for layer-by-layer construction of complex architectures [172]. Innovations include hybrid networks (e.g., PEGDA-GelMA) and AI-driven optimization for patient-specific implants, vascularized tissue constructs, and smart wound dressings [173].
- Kirigami Hydrogels: Laser-patterned thin films that swell into auxetic structures offer adaptive deformation and mechanical tunability. These are promising for soft robotics, flexible sensors, and intelligent materials [173].
- Nanocomposite Hydrogels: Integration of graphene, conductive polymers, and metal nanoparticles enhances electrical conductivity, mechanical strength, and stimuli responsiveness. These are being explored for energy devices, biosensors, and bioelectronics [174].
- Rheology-Guided Fabrication: Understanding viscoelastic properties (e.g., shear-thinning, thixotropy) is now central to optimizing printability and mechanical performance in bioprinting. Rheological profiling helps define the processing window for extrusion and inkjet printing [175].

These methods can be applied in both in situ crosslinking and post-synthetic crosslinking workflows, depending on the material system and desired film properties. Each technique offers unique advantages, but not all are universally compatible with every polymer type. Selecting the best method is challenging, as it depends on the film’s application, material properties, cost factors, and often simply on the equipment available to the researchers or organization. Table 2 shows a comparison of the main characteristics of these methods.

## 4. Characterization and Evaluation of Hydrogel Films

The characterization of hydrogel films is a critical step in understanding their physicochemical, mechanical, and biological properties, which directly influence their performance in biomedical applications. These evaluations ensure reproducibility, safety, and functionality, making them indispensable for research and clinical translation.

### 4.1. Spectroscopic Analysis

Spectroscopic techniques are fundamental for determining the chemical composition and molecular interactions within hydrogel networks. Fourier Transform Infrared (FTIR) spectroscopy is widely employed to identify functional groups and monitor crosslinking efficiency by analyzing characteristic absorption bands. Shifts in peak intensity or position often indicate chemical modifications during synthesis or environmental exposure, providing insights into the degree and type of crosslinking [178]. Raman spectroscopy complements FTIR by offering vibrational information on molecular structure and crystallinity, making it particularly useful for monitoring gelation processes and nanoparticle incorporation [179]. UV–Vis spectroscopy is applied to evaluate optical properties, transparency, and electronic transitions, and is frequently used to monitor self-healing kinetics and hydrophobicity changes in supramolecular hydrogels [180]. Nuclear Magnetic Resonance (NMR) spectroscopy provides detailed insights into polymer network architecture, water distribution, and molecular mobility. Solid-state NMR techniques, such as magic angle spinning (MAS), enhance resolution for semi-solid hydrogel systems, while proton relaxometry can differentiate between bound and free water populations [181]. Fluorescence spectroscopy is another powerful tool for probing microenvironmental heterogeneity and the dynamics of encapsulated species, enabling the visualization of network interactions and local polarity changes [182].

### 4.2. Thermal Analysis

Thermal characterization reveals phase transitions, thermal stability, and water–polymer interactions, which are crucial for predicting hydrogel performance under physiological and processing conditions. Differential Scanning Calorimetry (DSC) measures heat flow during temperature changes, detecting melting, crystallization, and glass transition events [178]. Thermogravimetric Analysis (TGA) quantifies weight loss during heating, providing insights into thermal stability and decomposition behavior [183]. Differential Thermal Analysis (DTA) compares temperature differences between a sample and a reference during heating or cooling, enabling the identification of phase transitions with high precision [184]. These techniques can be combined to reduce sample requirements and improve accuracy, offering a comprehensive understanding of hydrogel thermal behavior.

### 4.3. Mechanical Characterization

Mechanical properties determine the durability and functional reliability of hydrogel films in biomedical applications such as wound dressings, implant coatings, and tissue scaffolds. Key parameters include elastic modulus (Young’s modulus), tensile strength, fracture toughness, and viscoelasticity, which reflect stiffness, resistance to fracture, and time-dependent behavior such as stress relaxation and creep, respectively [185]. Tensile testing generates stress–strain curves for modulus and strength, while compression testing evaluates compressive strength and deformation under load. Indentation and nanoindentation techniques provide localized measurements of shear modulus, relaxation time, and water diffusion coefficients, offering insights into nanoscale mechanical behavior [186]. Additional evaluations for hydrogel films include scratch resistance, adhesion tests, and contact angle measurements to assess surface wettability and adhesion performance, which are critical for applications involving tissue contact or device integration.

### 4.4. Morphological and Microstructural Analysis

Morphological characterization provides essential information on surface architecture and internal structure, which influence adhesion, drug release, and cellular interactions. Optical microscopy offers initial structural observations, while confocal microscopy enables the three-dimensional reconstruction of hydrogel networks for detailed visualization of porosity and layer organization. Scanning Electron Microscopy (SEM) and Transmission Electron Microscopy (TEM) are widely used to examine nanoscale morphology and pore structure, with cryo-SEM and environmental SEM allowing hydrated-state imaging to preserve native hydrogel architecture [187]. Atomic Force Microscopy (AFM) is employed to measure surface topography, elastic modulus, and nanoscale adhesion properties, providing a comprehensive view of mechanical and structural features at the micro- and nanoscale.

### 4.5. Swelling and Water Uptake

Swelling tests quantify water absorption and network expansion, which are critical for drug delivery, tissue integration, and mechanical performance. Swelling behavior depends on polymer composition, crosslinking density, and environmental factors such as pH, temperature, and ionic strength. Hydrogels with higher crosslinking density typically exhibit lower swelling due to restricted network mobility, while hydrophilic functional groups enhance water uptake [180]. These tests provide insights into hydrogel responsiveness and stability under physiological conditions.

### 4.6. Permeability and Diffusion

Controlled permeability is essential for applications in drug delivery and biosensing. Franz diffusion cells are commonly used to measure drug permeation through hydrogel films, while fluorescent tracer studies visualize diffusion pathways and kinetics [188]. These evaluations enable the optimization of hydrogel formulations for sustained and targeted therapeutic release.

### 4.7. Degradation and Stability

Hydrogel degradation behavior is critical for applications requiring controlled resorption or long-term stability. Hydrolytic and enzymatic degradation studies provide insights into material breakdown under physiological conditions. Advanced imaging techniques such as micro-computed tomography (micro-CT) allow for the non-destructive visualization of structural integrity and porosity changes during oxidative stress, for example, after exposure to Fenton’s reagent [183].

**Scheme 2:**
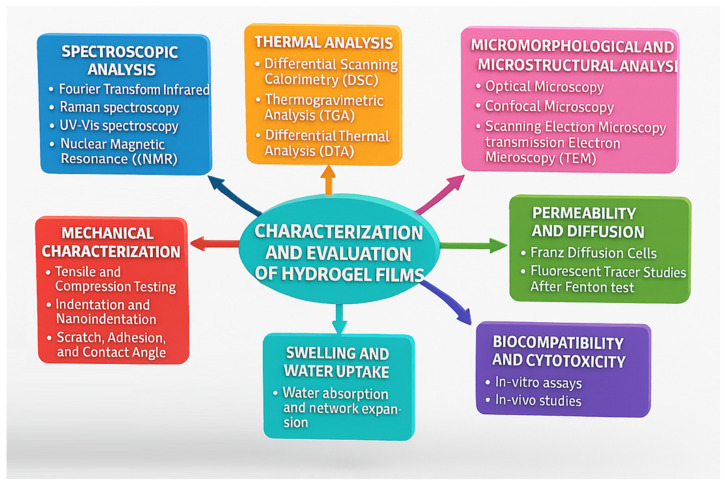
Overview of characterization and evaluation techniques for hydrogel films.

To ensure that hydrogel films meet the stringent requirements for biomedical applications, a wide range of characterization methods is employed. These techniques provide critical insights into chemical composition, structural integrity, thermal stability, mechanical performance, and biological compatibility. The following schematic summarizes the major categories of evaluation methods, including spectroscopic, thermal, mechanical, and morphological analyses, as well as swelling behavior, permeability, and biocompatibility assessments (Scheme 2).

## 5. Unique Properties of Hydrogel Films

Hydrogel films possess a distinctive combination of physicochemical and biological properties that make them highly suitable for biomedical applications. Their thin-film format, high water content, permeability control, and tissue-conforming behavior enable them to function effectively in wound healing, drug delivery, tissue engineering, and biosensing.

### 5.1. Thin-Film Architecture and Flexibility

Hydrogel thin films, typically fabricated with micrometer-scale thicknesses, have emerged as a versatile platform for biomedical applications due to their ability to conform intimately to soft, irregular biological surfaces. This thin-film architecture not only enhances tissue contact and drug absorption but also improves mechanical adaptability, making it ideal for applications requiring minimal invasiveness and high biocompatibility.

The flexibility of hydrogel films is largely attributed to their high water content and polymeric network structure, which mimics the extracellular matrix (ECM). These properties allow them to maintain softness, stretchability, and permeability, essential for dynamic biological environments. For instance, Gong et al. emphasized the importance of double-network hydrogels in achieving both toughness and flexibility, which has inspired the design of thin-film variants for wearable and implantable devices [185].

Recent advancements have focused on optimizing the mechanical strength of thin hydrogel films without compromising their flexibility. Nguyen et al. introduced a novel thin-film hydrogel (TFH) with a thickness of approximately 100 µm and 60% water content, synthesized via hydrophobic benzene–benzene interactions [186]. These TFHs exhibited remarkable mechanical properties, including a tensile strength of 2.35 MPa and a Young’s modulus of 4.7 MPa, along with excellent environmental stability, making them suitable for use as biological membranes and scaffolds. Moreover, thin-film hydrogels have been explored for ocular applications, such as corneal patches, where transparency, oxygen permeability, and conformability are critical. For example, Maulvi et al. (2016) developed a hydrogel-based contact lens for sustained drug delivery to the eye, demonstrating the potential of thin-film hydrogels in ophthalmology [189].

In transdermal drug delivery, thin hydrogel films serve as adhesive patches that can deliver therapeutic agents through the skin in a controlled manner. Their moisture-retaining and non-irritating nature makes them superior to traditional patches. Studies by Ngo et al. have shown that incorporating nanocarriers into hydrogel films can further enhance drug loading and release profiles [190].

Additionally, implant coatings made from hydrogel films can reduce foreign body responses and improve integration with host tissues. Their ability to be functionalized with bioactive molecules or antimicrobial agents adds another layer of utility in regenerative medicine and infection control.

### 5.2. High Water Content

Hydrogels are composed of three-dimensional, hydrophilic polymer networks capable of absorbing and retaining substantial amounts of water—often exceeding 90% by weight. This high water content closely mimics the extracellular matrix (ECM) of natural tissues, creating a moist, nutrient-rich, and mechanically compliant environment that supports cellular functions and tissue regeneration.

The porous and hydrated structure of hydrogels facilitates oxygen and nutrient diffusion, promotes cell adhesion and proliferation, and minimizes mechanical mismatch with soft tissues. These characteristics make hydrogels particularly suitable for applications in wound healing, tissue engineering, and implantable devices [191]. Moreover, the low interfacial tension between hydrogel surfaces and biological tissues reduces the risk of inflammation and immune rejection. Studies have shown that hydrogels can modulate the foreign body response by minimizing fibrotic encapsulation, especially when engineered with bioinert or bioactive surface chemistries [192,193]. Wei et al. further emphasized that the elastic moduli of many hydrogels are comparable to those of soft biological tissues (ranging from a few kPa to hundreds of kPa), which is critical for maintaining mechanical harmony with the host environment [194]. This mechanical compatibility is essential in applications such as drug delivery systems, where the hydrogel must deform with tissue movement, and in biological electrodes, where soft interfaces reduce tissue damage and improve signal fidelity. In addition, hydrogels can be functionalized with peptides, growth factors, or nanoparticles to enhance their bioactivity and targeted therapeutic performance. For example, Britton et al. developed a hydrogel with embedded exosomes for enhanced skin regeneration, demonstrating how water-rich matrices can serve as both structural and biochemical scaffolds [181].

Therefore, the high water content of hydrogels is not merely a structural feature but a biomimetic advantage that underpins their mechanical tunability and therapeutic potential across a wide range of biomedical applications.

### 5.3. Permeability and Diffusion Control

Hydrogel films are uniquely suited for biomedical applications due to their selective permeability and diffusion-regulating capabilities. Their hydrated polymer networks allow for the controlled transport of gases, nutrients, and therapeutic agents, which is essential for applications such as sustained drug release, biosensing, wound healing, and tissue regeneration.

The diffusion behavior in hydrogels is governed by several factors, including polymer composition, crosslinking density, pore size, and hydration level. These parameters can be finely tuned to achieve precise control over molecular transport, enabling the design of hydrogels that respond to specific physiological or environmental stimuli. Lavrentev et al. conducted a comprehensive review of diffusion-limited processes in hydrogels, emphasizing their role in drug encapsulation, nutrient delivery, and stimuli-responsive systems [180]. Their findings highlighted how denser crosslinking reduces pore size and slows diffusion, while loosely crosslinked networks allow for faster transport. This tunability is critical for designing time-controlled drug delivery systems and biosensors that require consistent analyte exchange.

In a complementary study, Kanduč et al. used molecular dynamics simulations to demonstrate that molecular shape, size, and chemistry significantly influence hydrogel permeability [179]. Their work revealed that collapsed hydrogel states, often induced by environmental triggers (e.g., pH, temperature, ionic strength), exhibit high selectivity by restricting the passage of larger or hydrophobic molecules. This property can be exploited to create smart hydrogels that release drugs only under specific conditions, enhancing therapeutic precision and minimizing side effects.

Furthermore, stimuli-responsive hydrogels—also known as “intelligent” or “smart” hydrogels—have been developed to dynamically alter their permeability in response to external cues. For example, thermo-responsive hydrogels based on poly(N-isopropylacrylamide) (PNIPAM) undergo a volume phase transition near body temperature, enabling on-demand drug release [128]. Similarly, pH-sensitive hydrogels have been used in gastrointestinal drug delivery, where they remain stable in the acidic stomach but swell and release their payload in the more neutral intestines [195].

In tissue engineering, oxygen and nutrient diffusion through hydrogel scaffolds is vital for maintaining cell viability and tissue integration. Hydrogels with gradient permeability or multi-layered structures have been engineered to mimic natural tissue interfaces, such as the skin or cornea, where different layers require distinct transport properties [196].

Hence, the permeability and diffusion control of hydrogel films are a cornerstone of their functionality in biomedical applications. Through careful design of their network architecture and responsive behavior, hydrogels can be tailored to meet the complex demands of controlled release, biosensing, and regenerative medicine.

### 5.4. Surface Adhesion and Conformability to Tissues

One of the most compelling features of hydrogel films is their ability to adhere to moist biological surfaces without the need for external adhesives. This intrinsic adhesion, combined with their softness and flexibility, allows hydrogel films to conform intimately to irregular tissue geometries, which is critical for enhancing therapeutic efficacy, sensor accuracy, and mechanical integration in biomedical applications.

Hydrogels achieve this conformability through their low elastic modulus, hydrated polymeric structure, and interfacial compatibility with biological tissues. These properties enable them to form tight, non-irritating interfaces with soft organs such as the brain, heart, lungs, and skin. The ability to maintain stable contact even under dynamic physiological conditions makes hydrogel films ideal for implantable devices, wound dressings, and bioelectronic interfaces. A notable example is the work by Chen et al., who developed a gelatin-based metamaterial hydrogel film with a tunable elastic modulus ranging from 20 to 420 kPa and a Poisson’s ratio from −0.25 to 0.52 [197]. These tunable mechanical properties allowed the hydrogel to match the mechanical behavior of ultra-soft tissues, such as the myocardium and pulmonary tissue. The films were successfully used to monitor cardiac deformation and respiratory signals, demonstrating their potential in implantable bioelectronics and real-time physiological monitoring.

In parallel, Bovone et al. reviewed advanced strategies for engineering hydrogel adhesion through chemical junction design, including covalent bonding, supramolecular interactions, and dynamic reversible bonds [198]. These approaches significantly enhance adhesion strength and durability, especially in wet and mechanically active environments. For instance, catechol-functionalized hydrogels, inspired by mussel adhesive proteins, have shown strong and reversible adhesion to wet tissues, making them promising candidates for surgical glues, biosensors, and wearable electronics [199]. Furthermore, bioinspired adhesion mechanisms—such as those mimicking gecko feet or octopus suckers—have been integrated into hydrogel designs to improve reusability, directional adhesion, and detachment control. These innovations are particularly valuable in soft robotics, flexible electronics, and dynamic tissue interfaces [200,201].

Therefore, the surface adhesion and conformability of hydrogel films are key enablers of their success in non-invasive, implantable, and wearable biomedical technologies. By tailoring their mechanical properties and interfacial chemistry, hydrogel films can be engineered to achieve stable, biocompatible, and functional integration with a wide range of biological tissues.

### 5.5. Biocompatibility of Hydrogel Films for Biomedical Applications

Hydrogel films are widely utilized in biomedical fields due to their intrinsic compatibility with biological systems. This compatibility stems from their soft mechanical behavior, hydrated polymeric architecture, and chemically tunable surfaces, which collectively support cellular interactions and tissue integration. Their three-dimensional polymer networks, formed from natural polymers like gelatin, alginate, and hyaluronic acid, or synthetic ones such as polyethylene glycol (PEG), polyvinyl alcohol (PVA), and polyacrylamide, allow precise control over physical and chemical properties. This versatility enables the tailoring of degradation rates, mechanical strength, and surface functionality to meet specific biomedical requirements [166,178].

Polymer selection, crosslinking methods, and surface functionalization are critical in enhancing hydrogel–tissue interactions. Hydrogels crosslinked via dynamic covalent bonds or supramolecular interactions show improved tissue adhesion and mechanical resilience while maintaining cytocompatibility [30,202,203,204]. In vitro studies using cell lines such as L929 fibroblasts and HeLa cells consistently report low cytotoxicity, while in vivo applications demonstrate minimal immune response and favorable tissue remodeling, especially when bioactive or anti-inflammatory agents are incorporated [205].

Hydrogels are tailored for specific biomedical applications. In drug delivery, they enable localized and sustained release of therapeutics, reducing systemic toxicity. For instance, PAM/CNT nanocomposite hydrogel films exhibit excellent cytocompatibility and hemocompatibility, making them effective carriers for doxorubicin in cancer therapy [43]. In tissue engineering, hydrogels act as scaffolds that support cell infiltration, angiogenesis, and matrix deposition, with tunable biodegradability and mechanical properties suited to regenerating tissues [32,206,207]. For implantable devices, hydrogel coatings reduce foreign body responses and enhance biointegration, as demonstrated by gelatin-based metamaterial hydrogels compatible with soft organs like the heart and lungs [208].

Recent advances focus on multifunctional hydrogels that combine biocompatibility with antibacterial, anti-inflammatory, or immunomodulatory properties [209,210,211]. Phenylboronic acid-modified chitosan hydrogels, for example, offer both cytocompatibility and antibacterial activity, making them ideal for wound healing [212]. Additionally, smart hydrogels responsive to environmental stimuli (e.g., pH, temperature, enzymes) are being developed for precision medicine, enabling adaptive tissue interaction and on-demand therapeutic delivery [213,214].

### 5.6. Biodegradability of Hydrogel Films for Biomedical Applications

Biodegradability is a critical property of hydrogel films used in biomedical applications such as tissue engineering, drug delivery, and wound healing [3,215,216,217]. Biodegradable hydrogels are designed to safely degrade within the body through enzymatic, hydrolytic, or stimuli-responsive mechanisms, eliminating the need for surgical removal and reducing long-term complications [1]. These materials align with tissue regeneration timelines or drug release schedules, improving therapeutic outcomes and patient compliance [218].

Hydrogel degradation occurs via hydrolysis (common in synthetic polymers like PLA, PGA, and PEG-based copolymers), enzymatic breakdown (typical in natural polymers such as gelatin, chitosan, alginate, and hyaluronic acid), or stimuli-responsive mechanisms triggered by pH, temperature, enzymes, light, or oxidative stress [219,220,221,222,223]. Design strategies for tunable biodegradability include adjusting crosslinking density, blending fast-degrading natural polymers with stable synthetic ones, and incorporating stimuli-responsive elements for spatiotemporal control over degradation and drug release [220,221].

Recent innovations include multi-layered hydrogel films with sequential degradation behavior, enabling phase-specific drug delivery. For example, a double-layer hydrogel system with curcumin-loaded chitosan nanoparticles and pirfenidone-encapsulated gelatin microspheres demonstrated synchronized degradation and drug release aligned with wound healing stages, promoting scar-free regeneration in vivo [224].

In drug delivery, biodegradable hydrogels act as temporary depots, releasing therapeutic agents over time and degrading into non-toxic byproducts, thereby enhancing bioavailability and minimizing side effects [225]. In tissue engineering, they serve as scaffolds that support cell infiltration, angiogenesis, and ECM deposition, then degrade to leave behind newly formed tissue [226]. In wound healing, biodegradable films maintain a moist environment, protect against infection, and degrade in synchrony with tissue repair, eliminating the need for dressing removal [224,227].

However, safety concerns remain. Partial degradation can produce toxic monomers or oligomers [228]. For instance, while PEG is generally safe, its monomer ethylene glycol is nephrotoxic and neurotoxic [229]. Short-chain degradation products may accumulate in organs or cross the blood–brain barrier, posing risks [230]. Therefore, evaluating the biocompatibility of degradation byproducts is essential, especially for long-term or implantable applications.

In summary, the biodegradability of hydrogel films is a key enabler of their success in biomedical applications. Through rational material design, stimuli-responsive engineering, and toxicity-aware formulation, hydrogel films can be tailored to degrade in harmony with therapeutic needs while ensuring safety, efficacy, and patient comfort [231,232].

## 6. Biomedical Applications of Hydrogel Films

In contemporary biomedical practice, hydrogel films serve a multifaceted role across a spectrum of applications encompassing wound dressings, sustained drug delivery systems, and scaffolds for tissue engineering. Their water absorption capabilities and biocompatibility allow hydrogel films to create optimal microenvironments for cell growth and regeneration [149]. Moreover, hydrogels facilitate the controlled release of therapeutic agents, enhancing treatment efficacy while reducing systemic side effects [233]. The versatility of these systems extends to complex wound care, including chronic and burn wounds, where traditional dressings often fall short. Compared to conventional materials, hydrogel films possess distinct advantages such as the ability to maintain moisture balance, conform to irregular wound surfaces, and provide enhanced patient comfort [234]. Innovations in polymer design and surface modification have further improved the integration of hydrogel films with biological tissues, minimizing foreign body reactions and promoting healing outcomes [235]. Looking ahead, the continual development of hydrogel films is anticipated to align with personalized medicine trends, incorporating smart, responsive materials that adapt dynamically to changes in the wound or tissue milieu, thereby offering tailored therapeutic interventions [236,237].

### 6.1. Wound Dressings: Moisture Retention, Antimicrobial, Anti-Inflammatory Incorporation, and Smart Monitoring

Hydrogel films have emerged as versatile platforms in wound care due to their ability to maintain a moist healing environment, deliver therapeutic agents, support tissue regeneration, and enable real-time monitoring. Their hydrophilic nature, biocompatibility, and tunable mechanical properties make them ideal for treating both acute and chronic wounds, including burns, diabetic ulcers, and pressure sores [238,239,240].

Maintaining optimal hydration at the wound site is critical for epithelialization, autolytic debridement, and minimizing scarring. Hydrogel films mimic the extracellular matrix (ECM), facilitating cell migration, proliferation, and angiogenesis [166,208,241]. Commercial products such as Healoderm and Intrasite Gel exemplify clinically successful hydrogel dressings that promote granulation and re-epithelialization while reducing pain and infection risk [242,243,244].

**Figure 3:**
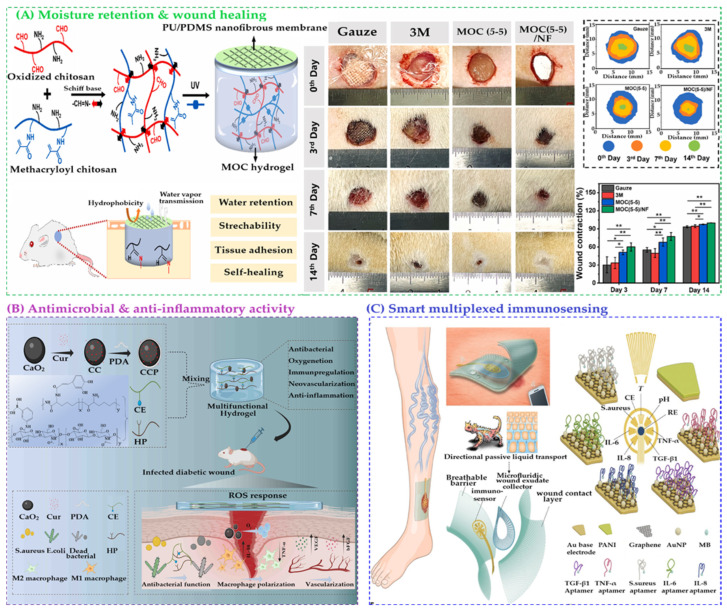
Schematic representation of the synthesis and applications of (**A**) a polyurethane/polydimethylsiloxane (PU/PDMS) nanofibrous (NF) membrane composite and methacrylated chitosan/oxidized chitosan (MOC (5–5))/NF hydrogel composites for wound healing (wound contraction rates. * *p* < 0.05, ** *p* < 0.01). Reproduced with permission from [245], Elsevier; (**B**) HP-CE@CCP hydrogel dressings, highlighting their antimicrobial and anti-inflammatory mechanisms in accelerating the healing of infected diabetic wounds. Reproduced with permission from [253], American Chemical Society. (**C**) Flexible microfluidic multiplexed immunosensing platform for point-of-care, in situ profiling of wound microenvironment, inflammation, and infection through multiplexed biomarker detection. Reproduced with permission from [254], American Association for the Advancement of Science.

Recent innovations have focused on hydrogel/nanofibrous membrane composites, which combine the moisture-retaining properties of hydrogels with the mechanical strength of nanofibers. Li et al. developed a bilayered PU/PDMS nanofibrous membrane with a self-healing chitosan-based hydrogel, showing enhanced stretchability and water retention (Figure 3A) [245]. Cheng et al. introduced a ZIF-8-encapsulated alginate hydrogel/polylactic acid nanofiber (CAH/PLANF) composite with photodynamic antibacterial properties and extracellular matrix (ECM)-like architecture, accelerating healing in infected wounds [246]. Ruan et al. further reviewed nanohybrid hydrogels integrating antibacterial agents, antioxidants, and stimuli-responsive drug delivery systems [247].

To address infection and inflammation—two major impediments to wound healing—hydrogel films have been engineered to incorporate antimicrobial agents, anti-inflammatory drugs, and natural bioactives. Ullah et al. developed a collagen-based hydrogel with Sr/Fe-substituted hydroxyapatite nanoparticles, ciprofloxacin, and dexamethasone, demonstrating antibacterial, anti-inflammatory, and osteogenic effects [248]. Pratinthong et al. modified CMC/PVA hydrogel films with citric acid and glutaraldehyde to enhance the anti-inflammatory efficacy of triamcinolone acetonide [249]. Xi et al. incorporated Fructus Ligustri Lucidi polysaccharide into PVA/pectin hydrogels, resulting in enhanced antibacterial activity, collagen deposition, and reduced inflammation [250].

Natural extracts have also been widely explored. Chuysinuan et al. formulated CMC/silk sericin hydrogel films with turmeric extract, showing strong antibacterial, antioxidant, and anti-inflammatory effects [251]. Fan et al. designed a multifunctional curcumin-loaded PVA/chitosan/sodium alginate hydrogel with potent antimicrobial, antioxidative, and angiogenic properties, promoting macrophage polarization and collagen synthesis in diabetic wound models [244]. Ahmady et al. embedded thymol-loaded alginate microparticles into chitosan-gelatin films, achieving controlled drug release, broad-spectrum antibacterial activity, and enhanced epithelialization in vivo [252].

Cadinoiu et al. created chitosan/PVA biocomposite films with silver nanoparticles and ibuprofen, showing synergistic antimicrobial and anti-inflammatory effects, validated through in vitro and in vivo studies [255]. Hashempur et al. developed a chitosan xerogel film with Nigella sativa extract using deep eutectic solvents, demonstrating strong antioxidant and antimicrobial activity against multiple pathogens [256].

To address chronic infected wounds, which are often exacerbated by persistent inflammation and reactive oxygen species (ROS), Tang et al. developed an ROS-responsive injectable hydrogel composed of ε-polylysine grafted with caffeic acid (EPL-CA) and hyaluronic acid grafted with phenylboronic acid (HP). The hydrogel was loaded with CaO_2_@Cur-PDA (CCP) nanoparticles, combining calcium peroxide (CaO_2_), curcumin (Cur), and polydopamine (PDA) (Figure 3B) [253]. Upon exposure to the wound microenvironment, the hydrogel gradually dissociates, enabling sequential release of therapeutic agents. Initially, caffeic acid-grafted ε-polylysine (CE) provides antibacterial and antioxidant effects, while hyaluronic acid (HA) mimics the extracellular matrix. Subsequently, CCP decomposes, releasing Cur, which promotes angiogenesis. This multi-phase release strategy aligns with the dynamic stages of wound healing, demonstrating effective bacterial clearance, ROS scavenging, and tissue regeneration in vivo. Boateng et al. formulated Polyox/carrageenan films loaded with streptomycin and diclofenac, achieving sustained drug release, high fluid absorption, and synergistic antibacterial and anti-inflammatory effects [257].

In addition to therapeutic functionalities, smart hydrogel films have been developed for real-time wound monitoring, particularly pH-responsive systems [258]. These dressings detect changes in wound pH—a key biomarker for infection and healing progression—and respond accordingly. Chronic wounds often exhibit elevated pH levels (above 7.0), indicating bacterial colonization and inflammation, while healing wounds maintain an acidic environment (pH 4.0–6.0) [259].

Wound pH is a reliable indicator of infection, with elevated levels (7.5–9.0) often signaling bacterial colonization and impaired healing. Gamerith et al. developed a silane-anchored bromocresol purple sensor that changes color from yellow to blue with increasing pH, enhancing visual contrast for infection detection [260]. Eskilson et al. improved spatial resolution using bacterial nanocellulose dressings embedded with mesoporous silica nanoparticles carrying pH-sensitive dyes, maintaining optimal wound dressing properties [261]. Electrochemical sensing offers real-time quantification. Rahimi et al. created a flexible pH sensor using laser-scribed ITO electrodes functionalized with polyaniline, achieving −55 mV/pH sensitivity across pH 4–10 and enabling wireless smartphone readout via NFC [262].

Hydrogel films have emerged as a promising platform for smart, multiplexed immunosensing in chronic wound care due to their biocompatibility, flexibility, and ability to interface directly with biological tissues. Chronic wounds, often caused by disrupted healing mechanisms, require continuous monitoring of multiple biomarkers to guide personalized treatment. Traditional diagnostic methods are limited in scope and accessibility, prompting the development of integrated biosensing systems. To address the complexity of chronic wounds, Gao et al. developed a graphene-based microfluidic immunosensor array that simultaneously detects tumor necrosis factor-α (TNF-α), interleukin-6 (IL-6), interleukin-8 (IL-8), and transforming growth factor–β1 (TGF-β1), *S. aureus*, pH, and temperature. Clinical trials showed a strong correlation between these biomarkers and delayed healing over five weeks (Figure 3C) [254]. A notable advancement is the creation of a flexible, hydrogel-based microfluidic platform capable of simultaneously detecting inflammatory cytokines (TNF-α, IL-6, IL-8, TGF-β1), microbial burden (*Staphylococcus aureus*), and physicochemical parameters (pH and temperature). This system, exemplified by the VeCare device, incorporates microdrop functionalization, novel aptamer sequences, and wireless electronics for real-time, smartphone-based data readout. The hydrogel film serves as both a sensing matrix and a wound interface, enabling in situ, point-of-care diagnostics. Clinical validation in animal models and human wound exudates demonstrates its potential to transform chronic wound management by enabling timely, personalized interventions and improving healing outcomes.

Han et al. categorized pH-responsive hydrogel mechanisms into morphological changes via dynamic bonds (e.g., Schiff base, catechol–Fe coordination), swelling behavior through protonation/deprotonation, degradation control using ester or imine bonds, and drug release modulation based on ion concentration and drug-polymer affinity [263]. These systems integrate optical indicators and electrochemical sensors, enabling color change or electrical signal generation in response to pH fluctuations. Representative studies include Mariani et al.’s textile-based smart bandage with a semiconducting polymer/IrOx pH sensor [264] and Kaewpradub et al.’s fully printed wearable bandage-based electrochemical sensor with pH correction [265]. Du et al. developed dual drug-loaded GelMA/HA-CHO hydrogels with the pH-responsive release of gentamicin and lysozyme [266], while Zhao et al. designed a multilayer hydrogel film (K-E-AGB) for diabetic wounds with glucose-responsive EGF release and early anti-inflammatory action [267]. Moeinipour et al. created a stimuli-responsive hydrogel film based on hydrogen-bonded organic frameworks (HOFs) for temperature and pH-triggered drug release [268].

These smart systems not only monitor the wound microenvironment but also actively respond to pathological changes, offering a personalized and proactive approach to wound care. Future directions include multi-parameter sensing (e.g., combining pH with temperature and bacterial load) [269], personalized therapy through responsive drug release [270], AI-guided analytics, and 3D bioprinting for customizable hydrogel architectures.

### 6.2. Hydrogel Films as Cell Culture

#### 6.2.1. Advanced Substrates for Cell Culture

Hydrogel films have gained prominence as dynamic platforms for cell culture due to their tunable physicochemical properties, biocompatibility, and ability to mimic the native extracellular matrix. Recent innovations in hydrogel design have focused on enhancing cellular interactions, spatial organization, and mechanical stability to support both mono- and co-culture systems.

One notable approach involves photocrosslinkable dextran-based hydrogel films, which utilize benzophenone-functionalized carboxymethyl dextran (BP-CMD) to form stable networks upon UV irradiation. These films can be reinforced with silica nanoparticles and gelatin microparticles, offering improved mechanical integrity and porosity. Importantly, the covalent immobilization of bioactive molecules such as BMP-2 enables the targeted stimulation of cell growth. This system has demonstrated excellent support for both osteoblast and endothelial cell proliferation, making it highly suitable for bone tissue engineering and vascularization studies [271].

Complementing this, surface-patterned hydrogel films present a versatile scaffold for 2D and 3D co-culture. By integrating magnetic silica rods on the hydrogel surface, these films allow for the spatial separation and simultaneous culture of different cell types—one embedded within the hydrogel matrix and another adhered to the patterned surface. This dual-architecture mimics tissue interfaces and facilitates the study of cell–cell interactions in a controlled microenvironment. The fabrication process is simple and scalable, offering potential for applications in organ-on-a-chip systems and multi-layered tissue constructs.

Together, these hydrogel film technologies represent a significant advancement in cell culture engineering. Their modularity, bioactivity, and spatial control capabilities make them promising tools for regenerative medicine, tissue modeling, and drug screening platforms [272].

**Figure 4:**
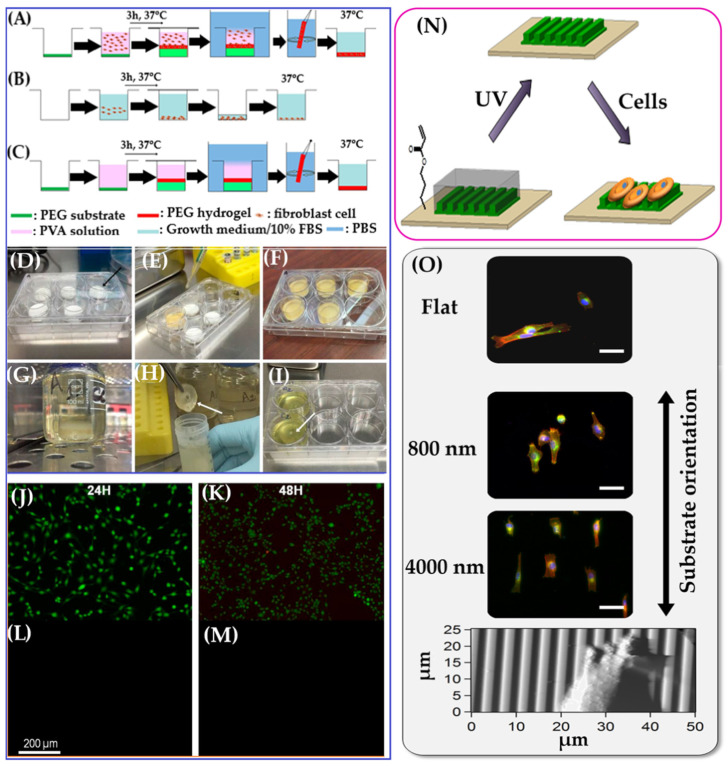
Schematic and experimental overview of encapsulation and control protocols. (**A**–**C**) Diagrams illustrate the encapsulation procedure (**A**), positive control for cell viability (**B**), and negative control for cell encapsulation (**C**). (**D**–**I**) Sequential steps of the encapsulation process: PEG substrates (black arrow) are placed in sterile glass wells (**D**); PVA/cell solution is added (**E**); incubation at 37 °C for 3 h (**F**); wells are soaked in excess growth medium (**G**); PVA hydrogel films (white arrow) are collected and rinsed in PBS (**H**); films (white arrow) are transferred to individual culture wells and incubated in growth medium at 37 °C (**I**). (**J**–**M**) Epifluorescence microscopy images of the positive control for cell viability (**J**,**K**) and the negative control for cell encapsulation (**L**,**M**) after 24 and 48 h, respectively. (**N**) Schematic of the fabrication process for topographically patterned PEG hydrogel films, where vertical swelling of molded features creates soft surface topography. (**O**) Fluorescence images of cells cultured on flat surfaces and on ridged/grooved substrates with 800 nm and 4000 nm pitch, alongside an AFM deflection image of a cell on a 4000 nm ridge/groove surface. Figure (**A**–**M**) reproduced with permission from [273], PANAS; (**N**,**O**) [274], American Chemical Society.

Hydrogels have a physical structure similar to the natural extracellular matrix, making them excellent materials for cell culture. A novel method was demonstrated by Moreau et al. for encapsulating cells in freestanding poly(vinyl alcohol) (PVA) hydrogel films, formed spontaneously through swelling-induced gelation. Mouse fibroblasts (NIH 3T3) were suspended in a PVA solution containing growth medium and fetal bovine serum, then poured into wells with dry, un-cross-linked PEG substrates. As the PEG swelled and dissolved, it triggered the formation of freestanding PVA hydrogel membranes (Figure 4A–M) [273].

This process minimized the need for direct handling and maintained aseptic conditions. The resulting films exhibited a thickness gradient (2 mm at the edges to 0.5 mm at the center). Epifluorescence imaging showed efficient cell encapsulation, with viability reaching ~70% for cells located more than 1 mm from the PEG interface after 24–48 h. Cells near the interface experienced reduced survival, likely due to hypertonic stress during swelling. This hydrogel environment offers a gentle, supportive, biocompatible, and scalable approach for in vitro cell culture applications.

#### 6.2.2. Hydrogel Films Mimicking Basement Membrane for Cell Culture

Hydrogel films designed to replicate the topography and mechanical compliance of the basement membrane offer a biologically relevant substrate for cell culture. These films aim to simulate the native microenvironment that cells experience in vivo, which is critical for maintaining physiological cell behavior.

Garland et al. developed a substrate with nano- to microscale surface features and tissue-like softness, closely resembling the basement membrane. This biomimetic design supports cell adhesion, spreading, and differentiation, particularly for epithelial and stem cells (Figure 4N,O) [274]. The substrate’s compliance and topographical cues were shown to influence cellular mechanotransduction, guiding cell morphology and function more effectively than conventional rigid culture surfaces. Such hydrogel-based substrates provide a more accurate platform for the in vitro modeling of tissue behavior, drug testing, and regenerative medicine, bridging the gap between traditional cell culture systems and the complexity of living tissues.

### 6.3. Drug Delivery Systems via Hydrogel Films

Hydrogel films have garnered substantial interest in drug delivery applications due to their unique combination of biocompatibility, high water content, and tunable physicochemical properties. These films, composed of three-dimensional hydrophilic polymer networks, can encapsulate a wide range of therapeutic agents—including small molecules, proteins, peptides, and nanoparticles—making them highly adaptable for various administration routes [275].

#### 6.3.1. Controlled and Sustained Release

One of the key advantages of hydrogel films is their ability to provide controlled and sustained drug release, which enhances therapeutic efficacy while minimizing systemic side effects. Hydrogel films offer precise control over drug release kinetics, maintaining therapeutic levels while minimizing dosing frequency and side effects [275]. Their ability to localize drug delivery reduces systemic toxicity, making them ideal for applications such as wound care and localized chemotherapy [276]. These systems also address challenges like burst release and premature drug degradation, ensuring a stable therapeutic window [166].

**Figure 5:**
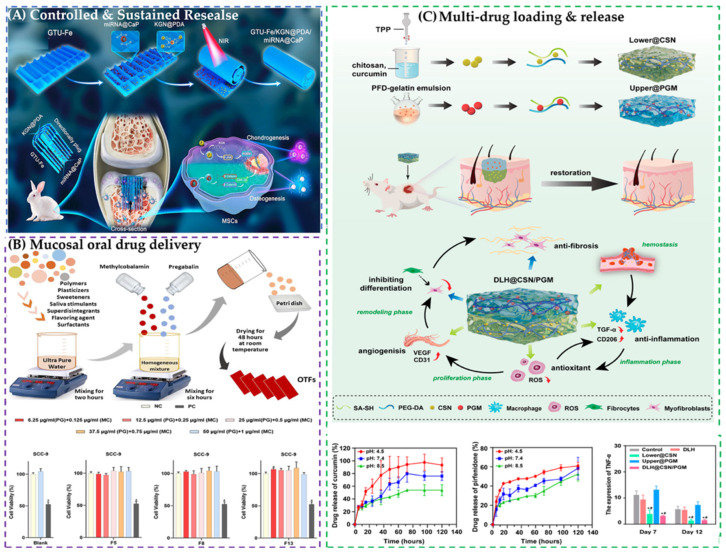
Multifunctional hydrogel film systems for targeted therapeutic applications. (**A**) Schematic illustrating the fabrication of oriented GTU-Fe/KGN@PDA/miRNA@CaP hydrogel film designed for osteochondral defect repair through controlled and sustained drug release. Reproduced with permission from [278], Springer Nature. (**B**) Preparation of oral thin films (OTFs) for mucosal drug delivery and evaluation of cell viability. Reproduced with permission from [279], MDPI. (**C**) Schematic of a double-layer hydrogel drug delivery system enabling multi-drug loading and sequential release to promote scar-free wound healing via multi-stage collaborative regulation (* *p* < 0.05 vs. Control; # *p* < 0.05 vs. DLH). Reproduced with permission from [224], MDPI.

The adjustable crosslinking and mesh size of hydrogels enable sustained release that is tailored to drug properties and clinical requirements [188]. For example, ciprofloxacin-loaded hydrogels demonstrated prolonged antibacterial activity and controlled release over 10 h, following first-order kinetics [277]. Additionally, effective osteochondral repair requires simultaneous regeneration of both cartilage and subchondral bone, which is often limited by single-agent delivery systems. To address this, Kang et al. developed a supramolecular hydrogel film capable of co-delivering two distinct therapeutic agents: kartogenin@polydopamine (KGN@PDA) nanoparticles for cartilage regeneration and miRNA@calcium phosphate (miRNA@CaP) nanoparticles for bone repair (Figure 5A) [278]. These agents were in situ deposited onto a patterned UPy-modified gelatin hydrogel via metal ion coordination, enabling spatially organized and targeted delivery. The hydrogel system supports controlled release of both KGN and miR-26a, promoting chondrogenic and osteogenic differentiation through the JNK/RUNX1 and GSK-3β/β-catenin pathways, respectively. In vivo, the cylindrical hydrogel plug mimicking the Haversian canal structure facilitated integrated regeneration of cartilage and bone, demonstrating enhanced tissue formation and functional restoration.

#### 6.3.2. Transdermal and Mucosal Delivery Platforms

Transdermal hydrogel patches exploit controlled permeability to deliver drugs systemically while bypassing gastrointestinal degradation and first-pass metabolism, enhancing bioavailability and patient adherence [280].

Abedini et al. developed dual-anionic hydrogel films using alginate and quince seed gum to deliver curcumin transdermally [281]. To improve compatibility with the hydrophilic matrix, curcumin was modified with stearic acid, enabling uniform dispersion and sustained release over 48 h. This system enhanced wound healing markers, demonstrating the potential of combining natural polymers and surface modification for effective transdermal delivery of hydrophobic drugs.

Mucosal films for oral and ocular drug delivery benefit from the adhesive, hydrating, and sustained release properties of hydrogel films, improving therapeutic outcomes in sensitive tissue environments [275]. The flexibility and conformability of such films greatly increase patient compliance, especially in chronic conditions requiring long-term treatment [166]. For example, to enhance the sublingual delivery of antifungal agents, researchers developed multilayered mucoadhesive hydrogel films incorporating nystatin [282]. The system combined Ocimum basilicum seed mucilage, thiolated alginate, and dopamine-modified hyaluronic acid, with a polydopamine (PDA) coating to improve adhesion and drug retention. This layered structure enabled the controlled and sustained release of nystatin, improving mucosal permeability and therapeutic efficacy. The study highlights the potential of multifunctional hydrogel films for the localized delivery of bioactive agents in oral applications.

Özakar et al. developed fast-dissolving hydrogel-based oral thin films incorporating pregabalin and methylcobalamin for improved management of neuropathic pain (Figure 5B) [279]. Designed for patients with swallowing difficulties, the films enable rapid disintegration and targeted delivery. The dual-drug system ensures the simultaneous release of both agents, enhancing therapeutic efficacy while maintaining biocompatibility and ease of administration. This approach highlights the potential of oral thin films for delivering multiple active compounds in a patient-friendly format.

#### 6.3.3. Hydrogel Film-Based Multi-Drug Loading and Release Kinetics

Hydrogel films have emerged as a promising platform for multi-drug delivery due to their high water content, biocompatibility, and tunable network structures. These properties enable controlled release profiles and spatial separation of therapeutic agents, which are critical for complex treatment regimens such as wound healing, cancer therapy, and immunosuppression.

Recent studies have explored diverse strategies for multi-drug incorporation. Yoon et al. reviewed hydrogel–nanoparticle composites that enable dual-drug delivery through physical embedding, covalent integration, and layer-by-layer assembly [283]. These designs allow for programmable, multi-phase release, enhancing therapeutic synergy and reducing side effects. Similarly, Manghnani et al. demonstrated how Michael addition-based PEG hydrogels can be chemically tuned to control the release of micro-crystalline fenofibrate [284]. By altering the crosslinking chemistry, the release duration was modulated from 4 h to 10 days, offering precise temporal control over drug availability.

Zhang et al. designed a biodegradable double-layer hydrogel film for scar-free wound healing, enabling multi-drug loading and sequential release kinetics (Figure 5C) [224]. The lower layer contained curcumin-loaded chitosan nanoparticles for early anti-inflammatory action, while the upper layer housed pirfenidone-encapsulated gelatin microspheres for delayed anti-fibrotic effects. The distinct degradation rates and mechanical properties of each layer facilitated phase-specific drug release, aligning with the wound healing stages. This controlled, time-resolved delivery strategy accelerated tissue regeneration and minimized scarring, demonstrating the hydrogel’s potential for multi-phase therapeutic regulation.

Hu et al. provided a comprehensive review of hydrogel drug delivery systems, emphasizing the role of polymer–drug interactions, crosslink density, and external stimuli (e.g., pH, temperature) in shaping release kinetics [188]. Their work highlights how hydrogels can be engineered to respond to physiological conditions, enabling site-specific and sustained drug release across various tissues.

Despite these advances, challenges remain in achieving reproducible multi-drug loading, precise spatial control, and predictive modeling of in vivo release behavior. Future research is expected to focus on integrating real-time monitoring systems and developing smart hydrogels capable of adaptive release in response to dynamic biological environments.

### 6.4. Tissue Engineering

Hydrogel films have garnered significant attention within tissue engineering due to their ability to closely mimic the native extracellular matrix (ECM), which is crucial for directing cellular behavior and tissue regeneration. The ECM is inherently hydrated and exhibits a porous, soft, and viscoelastic nature, providing both physical support and biochemical signaling to resident cells. Hydrogel films reproduce this soft, hydrated environment through their three-dimensional polymeric networks, offering a microenvironment conducive to promoting cell adhesion, proliferation, and differentiation. Notably, they ensure sufficient nutrient and gas exchange, necessary for cell viability and function, by virtue of their porous structure [101,149].

In contrast to traditional scaffolds made from rigid or synthetic materials, hydrogel films provide enhanced mechanical compliance, which better recapitulates the physiological stiffness of soft tissues, thereby minimizing foreign body reactions and fibrosis risks post-implantation. Their intrinsic biocompatibility allows for seamless integration with host tissues, enabling more effective tissue regeneration. These advantages not only aid in improving cell–matrix interactions but also facilitate the dynamic remodeling characteristic of natural tissues, thus reinforcing the utility of hydrogel films in regenerative medicine [285].

#### 6.4.1. Hydrogel Films as Barrier Layers in Wound Healing

Hydrogel films have become indispensable in tissue engineering due to their ability to mimic the extracellular matrix (ECM), support cellular functions, and serve as barrier layers or scaffolds for tissue regeneration. Their high water content, biocompatibility, and tunable mechanical properties make them ideal for both soft and hard tissue applications, including skin, bone, cartilage, and mucosal tissues.

Barrier-forming hydrogel films are designed to protect damaged tissues, prevent infection, and regulate biochemical exchange at wound or implant interfaces. These films act as bioadhesive interfaces, promoting tissue integration while preventing microbial infiltration and fluid loss. Recent innovations include Janus hydrogels, which feature asymmetric surfaces—one side optimized for adhesion and integration, the other for anti-fouling and protection [182]. These structures mimic natural biological barriers and have shown promise in skin and mucosal wound repair, hemostasis, and post-surgical sealing [286]. Biocompatible hydrogel films also demonstrated promising applications in tissue adhesives and sealants as a potential alternative or adjunct to sutures or staples in various clinical indications [287,288,289,290]. However, traditional adhesive hydrogels often struggle to stick to wet tissues and cannot prevent unwanted tissue adhesion after surgery.

**Figure 6:**
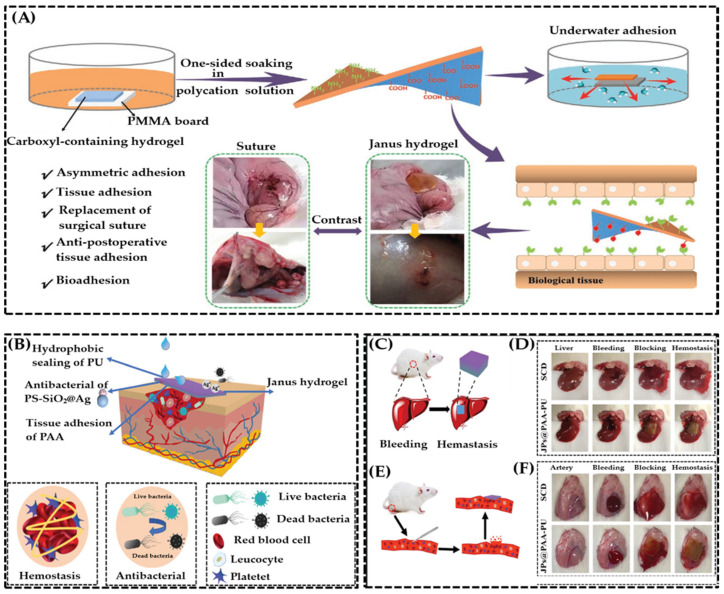
Design and biomedical application of the JPs@PAA-PU Janus hydrogel. (**A**) Schematic of Janus hydrogel formation via gradient polyelectrolyte complexation by dipping one side of the carboxyl-containing self-adhesive hydrogel into polycation solution; shown with applications in underwater adhesion and as a suture-free bandage for treating rabbit stomach perforation while preventing post-surgical adhesion. Reproduced with permission from [291], Copyright 2025, John Wiley and Sons. (**B**) Proposed hemostatic and antibacterial mechanism of the Janus hydrogel. (**C**) Schematic and (**D**) implementation of the rat-liver bleeding model demonstrating hemostatic performance. (**E**,**F**) Schematic and implementation of the rat-artery bleeding model. Reproduced with permission from [292], Copyright 2025, John Wiley and Sons.

To solve this problem, Cui et al. developed a Janus hydrogel—a film with two different surfaces: one sticky and one non-sticky (Figure 6A) [291]. This was achieved by dipping one side of a negatively charged hydrogel into a solution containing positively charged oligosaccharides. The dipping process created a gradient of electrostatic interactions, resulting in two distinct surfaces. The lightly treated side became highly adhesive, even underwater, due to increased hydrophobicity and better water drainage. It could strongly bond to wet tissues like pig skin, stomach, and intestine. In contrast, the heavily treated side became non-adhesive because the chemical groups responsible for sticking were neutralized. This design allowed the hydrogel to seal internal wounds while preventing external tissue from sticking, which is important for avoiding complications after surgery. In animal tests, the Janus hydrogel successfully repaired stomach perforations in rabbits and degraded naturally over 14 days. Its ability to heal itself in water and its safe interaction with tissues make it a promising material for future medical applications in internal tissue repair and anti-adhesion barriers.

#### 6.4.2. Hydrogel Films for Hemostasis and Anti-Adhesion in Wound Healing

Hydrogels are widely used in wound healing due to their ability to maintain a moist environment and adhere well to tissues, which helps stop bleeding and promote healing. However, traditional hydrogels with uniform structure can cause unwanted tissue adhesion, leading to secondary injuries. To overcome this, Fang et al. developed a Janus hydrogel with two distinct sides using a one-pot fabrication method. This hydrogel, called JPs@PAA-PU, is made from polyacrylic acid (PAA) and polyurushiol (PU), stabilized by special Janus particles. It has a water–oil layered structure without a separate adhesive layer, giving it unique physical and chemical properties (Figure 6B–F) [292]. The PAA side strongly adheres to tissues, red blood cells, and platelets, while the PU side supports blood clotting and acts as a physical barrier. This dual function allows the hydrogel to stop bleeding faster (as quickly as 32 s) and reduce blood loss compared to conventional materials. It also shows strong antibacterial activity against common bacteria like *E. coli* and *Staphylococcus aureus*, and has been proven safe for clinical use. The hydrogel’s asymmetric toughness—with higher strength on the adhesive side—further supports its role in targeted tissue interaction. Overall, this Janus hydrogel offers a promising solution for rapid hemostasis, infection control, and the prevention of tissue adhesion in wound care and surgical applications.

Hydrogel barriers are also being explored for implant coatings, where they reduce immune response and enhance biocompatibility. For example, nanocellulose-based hydrogels have demonstrated excellent mechanical strength and bioactivity, making them suitable for barrier applications in bone and vascular implants [293].

#### 6.4.3. Structural Scaffolding & Mechanical Reinforcement

Hydrogel films act as structural scaffolds that provide mechanical support for cell growth and tissue formation. These scaffolds can be engineered from natural polymers (e.g., collagen, gelatin, alginate, chitosan) or synthetic polymers (e.g., PEG, PVA), often in hybrid combinations to balance biological activity and mechanical resilience [226,294]. Their mechanical properties can be finely tuned through polymer selection, crosslinking density, and incorporation of reinforcing composite materials to closely approximate native tissue stiffness [295]. These adjustments ensure that scaffolds can sustain physiological loads and accommodate cellular remodeling without premature failure.

Nanofillers such as cellulose nanocrystals and silver nanoparticles impart enhanced mechanical strength and toughness while simultaneously contributing antimicrobial properties that protect against infection during tissue regeneration [296,297]. Additionally, design strategies incorporating anisotropic and hierarchical architectures emulate native tissue organization, improving functional outcomes by directing cell alignment and extracellular matrix deposition [187]. In advanced wound care, hydrogel films serve not only as drug carriers but also as structural scaffolds that support tissue regeneration. A recent study introduced a self-crosslinked chitosan (CS) hydrogel film reinforced with oxidized cellulose nanocrystal–silver nanoparticles (CNC-AgNPs), which stabilized a Pickering emulsion (PE) for delivering quercetin (Qu) [296]. This design enabled the formation of a robust interpenetrated network through Schiff base bonding between aldehyde groups of CNC-AgNPs and amino groups of CS, enhancing the film’s mechanical integrity. The hydrogel demonstrated excellent biocompatibility, non-hemolytic behavior, and promoted cell migration and collagen synthesis, crucial for full-thickness wound healing. In vivo studies confirmed accelerated wound closure and tissue regeneration, positioning this multifunctional hydrogel as a promising scaffold for clinical applications in skin repair.

**Figure 7:**
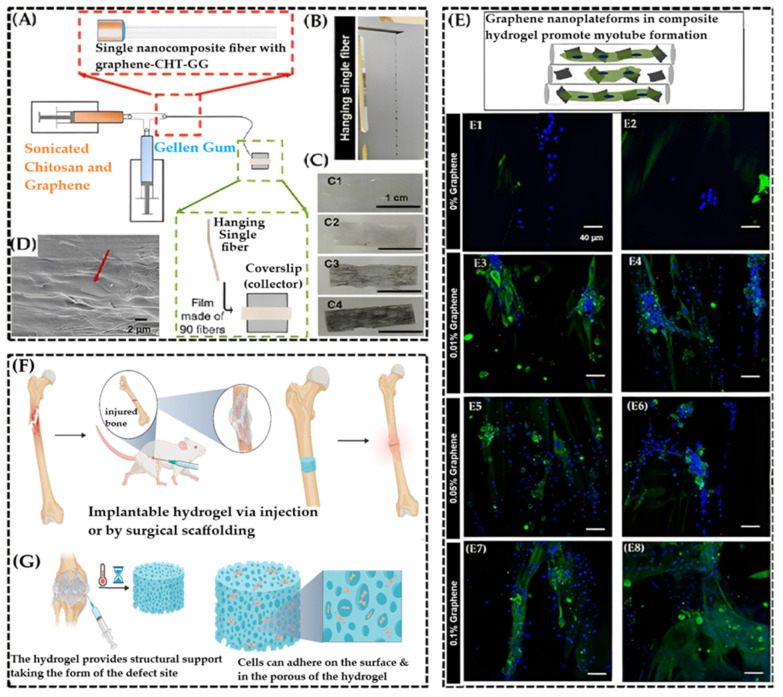
Fabrication and bioactivity of graphene–chitosan (CHT)–gellan gum (GG) nanocomposite films. (**A**) Schematic of microfluidic fiber fabrication setup; red and green boxes highlight fiber formation and collection zones. (**B**) Single fiber at needle tip. (**C1**–**C4**) Photos of control and graphene-loaded films (0.01%, 0.05%, 0.1%); scale bars = 1 cm. (**D**) SEM image showing surface-oriented graphene sheet (red arrow). (**E**) C2C12 myoblast differentiation on films; confocal images show Hoechst-stained nuclei (blue) and MHC-positive myotubes (green) with increasing graphene content (**E1**–**E8**), Reproduced with permission from [187], WILEY. (**F**) Hydrogel implantation via injection or scaffolding at injury sites. (**G**) Stimuli-responsive hydrogel adapts to defect shape, enhancing bone adhesion and cell proliferation. Reproduced with permission from [298], MDPI.

Biomaterial-based scaffolds play a critical role in enhancing cell survival and functional maturation in skeletal muscle regeneration. Nanocomposite fibrous hydrogel films incorporating graphene have been shown to support this process by providing anisotropic, bioactive scaffolds that mimic the native muscle extracellular matrix. Patel et al. developed hierarchically aligned fibrous hydrogel films using a microfluidic self-assembly technique that combines graphene with natural polysaccharides. In this context, when C2C12 myoblasts were cultured on these films, they initially formed aggregates and later differentiated into myotubes under myogenic conditions (Figure 7A–E) [187]. At lower graphene concentrations (0.01% and 0.05%), cells tended to form dense, rounded aggregates with the positive expression of myosin heavy chain (MHC), indicating early myogenic activity. In contrast, films with 0.1% graphene promoted the formation of elongated, multinucleated myotubes aligned along the fiber direction, suggesting enhanced and directional myogenesis. This transition from aggregation to alignment is attributed to the synergistic effects of graphene-induced nanoroughness, increased electrical conductivity, and optimal surface wettability. These properties collectively enhance cell–matrix interactions, promote cell spreading and fusion, and support the structural and functional maturation of muscle tissue. Such nanocomposite hydrogels offer a promising platform for skeletal muscle regeneration by combining mechanical reinforcement with bioinstructive cues.

Recent studies have emphasized the importance of porosity, degradation rate, and mechanotransduction in scaffold design. For instance, gelatin methacryloyl (GelMA) granular hydrogel scaffolds with macropores have shown enhanced macrophage-mediated healing and reduced inflammation in full-thickness skin wounds [6]. Similarly, injectable hydrogel scaffolds have been developed for cartilage regeneration, offering minimally invasive delivery and the controlled release of bioactive agents [299,300].

In bone tissue engineering, hydrogel scaffolds are being used to deliver osteogenic cells and growth factors, promote vascularization, and support mineralization. Studies have shown that hydrogel-based systems can effectively mimic the bone microenvironment and facilitate osteoblast differentiation, especially when combined with bioactive ceramics or nanoparticles [301].

Hydrogels have emerged as a promising class of biomaterials in bone tissue engineering (BTE) due to their ability to mimic the extracellular matrix and facilitate osteogenic drug delivery (Figure 7F) [298]. Their polymeric networks, particularly those derived from synthetic and natural biomacromolecules, offer inherent biocompatibility and tunable biofunctionality. Recent developments have emphasized hydrogels fabricated from biomacromolecules to enhance tissue integration and reduce inflammatory responses, eliminating the need for surgical removal in case of failure. These materials can be engineered for specific geometries suitable for implantation or injection, with controlled degradation rates, porosity, and drug release profiles achieved through tailored cross-linking strategies. Importantly, hydrogels provide structural support while creating a conducive microenvironment for bone regeneration. They enable osteoblast adhesion both on the surface and within the porous matrix, promoting cell proliferation, differentiation, and maturation—key processes in effective bone healing.

### 6.5. Ophthalmic Applications

Hydrogel films have revolutionized ophthalmic applications due to their exceptional biocompatibility, optical transparency, and moisture-retention capabilities, which are critical for maintaining ocular surface integrity and comfort. Their ability to conform to the complex anatomy of the eye and respond to physiological stimuli makes them ideal for a wide range of therapeutic and diagnostic uses, including contact lenses, corneal patches, and ocular drug delivery systems [302].

#### 6.5.1. Contact Lenses and Corneal Patches

Hydrogel films have become foundational materials in ophthalmology, particularly in the development of soft contact lenses and corneal patches, due to their biocompatibility, optical clarity, moisture retention, and oxygen permeability. These properties allow hydrogel films to mimic the natural hydration and mechanical environment of the cornea, making them ideal for both vision correction and therapeutic applications. Hydrogels such as poly(2-hydroxyethyl methacrylate) (pHEMA), polyvinyl alcohol (PVA), and silicone-based hydrogels are widely used in soft contact lenses. Their hydrophilic nature enables high water content, which enhances comfort and reduces friction with the ocular surface [303]. Recent advances include copolymer hydrogel systems that improve drug loading capacity and release kinetics, enabling contact lenses to serve as ocular drug delivery platforms [304].

**Figure 8:**
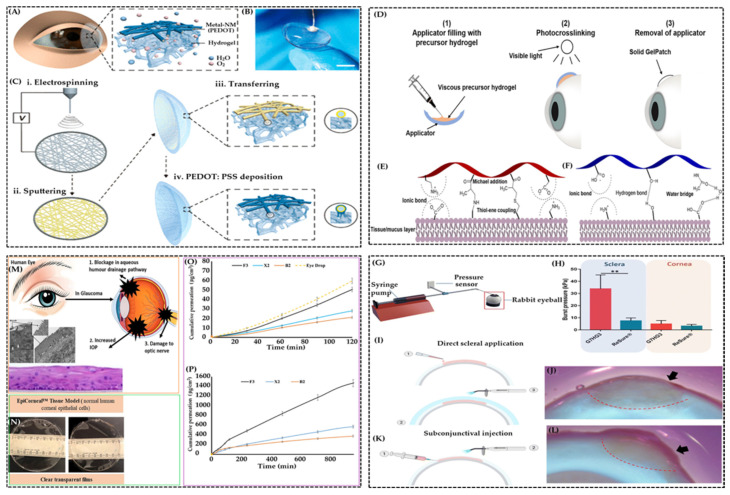
Design, application, and drug delivery performance of hydrogel-based ocular platforms. (**A**–**C**) Synthesis and structure of a gas-permeable, irritation-free, transparent hydrogel contact lens based on Auc-NM and electrochemically deposited PEDOT/PSS: (**A**) schematic of device structure, (**B**) digital photograph, and (**C**) fabrication steps. Reproduced with permission from [305], ACS. (**D**–**F**) GelPatch application and adhesion mechanism: (**D**) stepwise application using a contact lens applicator and visible light photocrosslinking, (**E**,**F**) schematic of chemical and physical bonding between GelMA/HAGM polymer chains and ocular tissue. (**G**,**H**) Ex vivo adhesion and retention assessment: (**G**) burst pressure setup using rabbit eyeball, (**H**) comparison of burst pressure between G7HG3 GelPatch and ReSure^®^ sealant. (**I**–**L**) GelPatch delivery routes: (**I**,**J**) direct scleral application with contact lens overlay, (**K**,**L**) subconjunctival injection and crosslinking, Reproduced with permission from [306], ACS. (**M**–**P**) Drug delivery evaluation: (**M**) schematic of glaucoma-affected eye, (**N**) visual transparency of drug-loaded HA crosslinked film (X2) and bilayer pHEMA/PVP-HA film (B2), (**O**) in vitro cumulative permeation of Timolol Maleate (TM) using EpiCorneal™ tissue, and (**P**) ex vivo permeation using porcine cornea. Data shown as mean (n = 2, ± SD) (** *p* < 0.01). Reproduced with permission from [307], Elsevier.

Wei et al. developed smart contact lenses featuring a highly porous, gas-permeable, and optically transparent design using a metal-coated nanofiber mesh (metalc-NM) (Figure 8A–C) [305]. Their fabrication process involved sputtering a thin layer of gold (~110 nm) onto electrospun polyacrylonitrile (PAN) nanofibers, which were then integrated with commercial hydrogel-based disposable contact lenses to form a composite structure. To enhance adhesion between the metal layer and the substrate, an electrochemical deposition of poly(3,4-ethylenedioxythiophene):poly(styrene sulfonate) (PEDOT:PSS) was applied. The resulting smart lenses demonstrated excellent gas permeability, hydration, wettability, optical clarity, and mechanical durability, making them suitable for advanced wearable biomedical applications.

Innovative designs such as microfluidic hydrogel-embedded contact lenses have demonstrated pH-responsive drug release, allowing for on-demand delivery of therapeutics in response to ocular conditions like inflammation [308]. These lenses also offer potential for real-time diagnostics, such as intraocular pressure monitoring and tear fluid analysis [309]

Hydrogel films are also being developed as corneal patches for wound healing, post-surgical repair, and tissue regeneration. Their ability to adhere to the corneal surface without sutures makes them attractive for minimally invasive treatments. For example, adhesive hydrogels composed of oxidized guar gum and carboxymethyl chitosan have shown self-healing, injectable, and tissue-adhesive properties, promoting corneal regeneration in rabbit models [310].

GelPatch, composed of gelatin methacryloyl (GelMA) and glycidyl methacrylated hyaluronic acid (HAGM), is a photocrosslinkable adhesive hydrogel film engineered for ocular tissue sealing (Figure 8D–L) [306]. Designed to address laceration-type injuries, it exhibits high burst pressure, minimal swelling, and strong adhesion to both scleral and subconjunctival tissues. These properties enable rapid and effective wound closure without sutures, positioning GelPatch as a promising candidate for sutureless ocular repair.

Another promising material, GelCORE, a light-crosslinkable bioadhesive hydrogel, has demonstrated superior tissue adhesion, transparency, and stromal regeneration compared to commercial adhesives [288]. These hydrogel patches can be tailored to match the geometry of corneal defects and support epithelial and stromal healing.

#### 6.5.2. Regenerative and Bioactive Potential

Hydrogel films can be functionalized with stem cells, extracellular vesicles, and bioactive molecules to enhance their regenerative capabilities. For instance, MSC-exosome-loaded hydrogels have been shown to promote collagen deposition, reduce inflammation, and accelerate healing in corneal injuries [310]. Additionally, heparin-functionalized hydrogels have been designed to sequester inflammatory cytokines and prevent fibrosis, offering a pathway to scarless corneal repair [311]. The integration of 3D bioprinting, stimuli-responsive polymers, and bioelectronic interfaces is expected to further advance hydrogel film technologies in ophthalmology. These innovations aim to create multifunctional platforms that combine drug delivery, diagnostics, and regenerative therapy in a single device.

Hydrogel films in contact lenses and corneal patches are not only improving patient comfort and compliance, but also redefining the possibilities for non-invasive ocular treatment and vision restoration.

### 6.6. Ocular Drug Delivery Films

Hydrogel films have emerged as a transformative solution for ocular drug delivery, addressing the limitations of conventional eye drops and ointments, which suffer from low bioavailability, rapid clearance, and poor patient compliance. Their high water content, biocompatibility, and mucoadhesive properties make them ideal for sustained, localized, and non-invasive drug release directly at the ocular surface [312].

#### 6.6.1. Challenges in Conventional Ocular Delivery

The eye’s unique anatomy—including the corneal epithelium, blinking reflex, and nasolacrimal drainage system—poses significant barriers to drug absorption. Less than 5% of topically administered drugs typically reach intraocular tissues [313]. Hydrogel films overcome these challenges by forming a protective, drug-loaded matrix that adheres to the ocular surface and releases medication over extended periods [312].

#### 6.6.2. Hydrogel Film Technologies

Hydrogel films for ocular drug delivery are fabricated using natural polymers (e.g., hyaluronic acid, chitosan, alginate) and synthetic polymers (e.g., polyvinyl alcohol, polyacrylamide, PEG derivatives). These materials can be engineered to respond to physiological stimuli such as pH, temperature, or ionic strength, enabling in situ gelation and on-demand drug release [312,314].

Tighsazzadeh et al. explored the development of hydrogel films composed of matrix hyaluronic acid (HA) and bilayer poly-hydroxyethyl methacrylate (pHEMA)-HA as innovative platforms for ocular drug delivery (Figure 8M–P) [307]. These films combine the biocompatibility and hydration properties of HA with the mechanical stability and permeability control of pHEMA, forming a layered structure suitable for sustained drug release. The bilayer design allows for modulated diffusion, enhancing therapeutic retention on the ocular surface while maintaining optical transparency and comfort. Such hydrogel films offer a promising alternative to conventional eye drops by improving bioavailability, residence time, and patient compliance in treating ocular conditions.

Recent advances include microfluidic hydrogel films embedded in contact lenses, which release drugs in response to ocular pH changes. For example, pH-responsive hydrogel microcavities have been shown to accelerate drug release under acidic conditions associated with inflammation [309].

#### 6.6.3. Nanoparticle-Enhanced Hydrogel Films

The integration of nanoparticles into hydrogel films has further enhanced their drug delivery capabilities. These hydrogel–nanoparticle composites allow for dual-drug loading, targeted delivery, and programmable release kinetics. A 2024 review by Arabpour et al. highlighted the use of nanosuspensions and nanoemulsions within hydrogel matrices to treat conditions such as glaucoma, dry eye disease, and retinal disorders [315]. These systems improve drug stability and enable the delivery of large molecules and biologics to both anterior and posterior segments of the eye, overcoming anatomical barriers such as the blood–retinal barrier [316].

#### 6.6.4. Clinical Applications and Innovations

Hydrogel films are being developed for a wide range of ophthalmic conditions:

- Dry eye syndrome: Films loaded with lubricants and anti-inflammatory agents [312].
- Glaucoma: Sustained release of prostaglandin analogs to reduce intraocular pressure [315].
- Post-surgical care: Antibiotic-loaded films to prevent infection and promote healing [317].
- Retinal diseases: Intravitreal hydrogel implants for long-term drug delivery [316].

Commercial formulations are beginning to emerge. For instance, CsA-PG ophthalmic gel, a cyclosporine-loaded hydrogel, is currently in Phase III clinical trials for treating moderate to severe dry eye disease, showing promising results in reducing inflammation and improving tear production [318].

The future of ocular drug delivery films lies in the development of multifunctional, stimuli-responsive systems that combine therapeutics, diagnostics, and regenerative capabilities. Promising directions include:

- Bioelectronic hydrogel films for real-time monitoring and feedback-controlled release;
- Personalized hydrogel formulations using AI-guided design and bioprinting;
- Stem cell and exosome-loaded hydrogel films for regenerative ophthalmology [309].

Despite their promise, most hydrogel-based ocular drug delivery systems remain in preclinical stages, and further clinical validation is needed to ensure safety, efficacy, and scalability [312].

### 6.7. Implant Coatings and Biosensors

Hydrogel films have gained significant attention in biomedical engineering as implant coatings and biosensing interfaces due to their biocompatibility, mechanical tunability, and functional versatility [319]. Their ability to mimic the extracellular matrix (ECM), retain moisture, and respond to physiological stimuli makes them ideal for enhancing implant integration, reducing immune response, and enabling real-time diagnostics [320].

#### 6.7.1. Implant Coatings

Hydrogel films are increasingly used as surface coatings for medical implants, including orthopedic devices, neural interfaces, and cardiovascular implants. Their soft, hydrated nature reduces mechanical mismatch between the implant and surrounding tissue, thereby minimizing foreign body reactions (FBR) and fibrotic encapsulation [321].

Recent studies have shown that hydrogels with elastic moduli below 1 kPa can suppress the activation of mechanosensitive cells such as astrocytes and glia, promoting long-term biointegration in neural implants [321]. These coatings can also be engineered to release anti-inflammatory agents, antibiotics, or growth factors, enhancing tissue healing and preventing infection. Advanced hydrogel coatings incorporate conductive polymers (e.g., PEDOT, polypyrrole) or nanomaterials (e.g., carbon nanotubes, graphene) to create electrically conductive hydrogels, which facilitate charge transfer in bioelectronic implants while maintaining mechanical compliance [321]. Such coatings are particularly valuable in neural stimulation, cardiac pacing, and biosignal recording. Moreover, antibacterial hydrogel coatings have been developed to combat implant-associated infections, especially in orthopedic applications. These coatings utilize strategies such as contact killing, biofilm disruption, and drug delivery to prevent bacterial colonization and enhance implant longevity [322].

**Figure 9:**
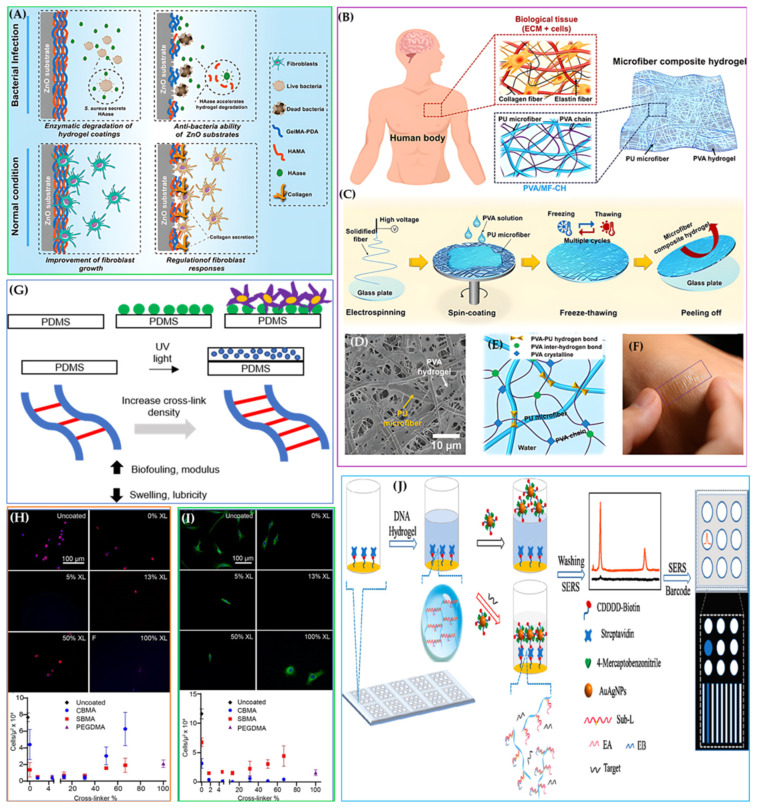
Schematic overview of the self-adaptive strategy and bioelectronic design. (**A**) Enzymatically degradable hydrogel coatings on titanium for bacterial inhibition and enhanced soft tissue compatibility, Reproduced with permission from [323], Elsevier. (**B**) ECM-mimicking structure of PVA/MF-CH, composed of interpenetrating PU microfibers (blue) and PVA chains (purple) within a semi-transparent hydrogel matrix. (**C**) Preparation process of PVA/MF-CH. (**D**) SEM image of freeze-dried PVA/MF-CH surface. (**E**) Illustration of microfiber embedding and bonding mechanisms between PU matrix and PVA chains. (**F**) Photograph of PVA/MF-CH applied to skin, Reproduced with permission from [324], SpringerNature. (**G**) Schematic showing the influence of PEGDMA cross-linker concentration on protein adsorption, cell adhesion, swelling, compressive modulus, and lubricity of zwitterionic thin films. (**H**) Macrophage adhesion on uncoated PDMS and CBMA-coated films with varying PEGDMA levels, visualized using anti-F4/8 (red) and DAPI (blue). (**I**) Fibroblast adhesion on uncoated PDMS and CBMA-coated films with varying PEGDMA levels, visualized using antivimentin (green) and DAPI (blue), reprinted with permission from [325], ACS. (**J**) Schematic illustration of the synthesis and functional deployment of a target miRNA-responsive DNA hydrogel-based SERS sensor array, designed for simultaneous detection of multiple miRNAs within a single biological sample, reprinted with permission from [326], ACS.

Hydrogel films have emerged as promising coatings for titanium implants, addressing key challenges such as bacterial infection and poor soft tissue integration. In a recent study, an enzymatically degradable hydrogel was engineered to cover titanium surfaces, offering a self-adaptive response to infection (Figure 9A) [323]. Under normal conditions, the hydrogel promotes fibroblast viability and soft tissue compatibility. Upon bacterial invasion, the hydrogel degrades, exposing an underlying ZnO nanostructure that activates antibacterial effects. This dual-function system enhances implant safety and healing by combining infection control with tissue regeneration, demonstrating the potential of smart hydrogel coatings in biomedical implants.

#### 6.7.2. Hydrogel Films Integrated Biosensors

Hydrogel films are also being integrated into biosensors for diagnostics, wearable health monitoring, and implantable bioelectronics. Their porous structure, high water content, and biomolecule immobilization capacity make them ideal for detecting analytes such as glucose, lactate, urea, and pathogens [327].

Hydrogel films are ideal for implantable biosensors due to their high biocompatibility, flexibility, and tissue-like mechanical properties. A recent study by Gao et al. introduced a microfiber composite hydrogel (MF-CH), integrating electrospun polyurethane (PU) microfibers into a poly(vinyl alcohol) (PVA) matrix (Figure 9B–F) [324]. This design achieved an ultrasoft, ultrathin (<5 μm) and mechanically robust structure, mimicking the extracellular matrix (ECM). The MF-CH exhibited high tensile strength (~6 MPa), tunable modulus (5 kPa–tens of MPa), and excellent anti-tearing properties. Enhanced with glycerol and ionic salts, the hydrogel demonstrated improved ionic conductivity and dehydration resistance, ensuring stable performance in physiological environments. Its application in electromyography (EMG) sensing confirmed its potential as a high-fidelity, long-term bioelectronic interface. These findings position MF-CHs as promising candidates for next-generation implantable biosensors, offering seamless tissue integration and reliable signal acquisition.

Hydrogel-based biosensors can be functionalized with enzymes, antibodies, or aptamers, enabling high specificity and sensitivity. These sensors are used in applications ranging from point-of-care diagnostics to continuous monitoring of chronic diseases. Recent innovations include electrochemical hydrogel biosensors embedded in wearable devices, capable of real-time monitoring of physiological parameters such as sweat composition, temperature, and strain [328]. Conductive hydrogels with nanocomposite fillers have enabled the development of flexible, stretchable, and self-healing biosensors, suitable for electronic skin and prosthetics [329].

The future of hydrogel films in implant coatings and biosensors lies in the development of multifunctional, smart materials that combine therapeutic, diagnostic, and regenerative capabilities. Promising directions include:

- Self-healing hydrogel coatings for long-term implant durability;
- Stimuli-responsive biosensors for dynamic health monitoring;
- 3D-printed hydrogel interfaces for personalized implant design;
- Bioelectronic hydrogel platforms for integrated sensing and stimulation.

These innovations are paving the way for next-generation biomedical devices that are minimally invasive, highly adaptive, and clinically effective.

### 6.8. Anti-Fouling and Biointegration

Hydrogel films are increasingly recognized for their dual role in preventing biofouling and promoting biointegration in biomedical devices and implants. Their hydrated, soft, and tunable surface chemistry allows them to mimic the extracellular matrix (ECM), reduce immune responses, and resist nonspecific protein adsorption and microbial colonization—two critical factors in long-term implant success.

#### 6.8.1. Anti-Fouling Properties

Biofouling begins with the adsorption of proteins and biomolecules onto implant surfaces, which can lead to bacterial colonization, biofilm formation, and chronic inflammation [330]. Hydrogel films, particularly those based on zwitterionic polymers such as poly(sulfobetaine methacrylate) (pSBMA), have shown excellent resistance to protein and cell adhesion due to their strong hydration layers. These coatings can be further enhanced by incorporating cationic bactericidal polymers, creating dual-functional surfaces that both repel and kill bacteria like *E. coli* and *S. aureus* [331].

Zwitterionic hydrogel films have gained attention for implantable applications due to their exceptional antifouling properties and tunable mechanical performance. By photografting zwitterionic polymers onto substrates like PDMS, researchers achieved up to a 20-fold reduction in fibrinogen adsorption and significantly decreased macrophage (30-fold) and fibroblast (10-fold) adhesion, minimizing foreign body response (Figure 9G–I) [325]. Cross-linking with PEGDMA modulates swelling, compressive modulus, and lubricity, with optimal densities balancing mechanical integrity and biological inertness. Notably, these films exhibit a lower coefficient of friction, enhancing suitability for insertional implants. Overall, zwitterionic hydrogels offer a robust platform for bioinert, durable, and lubricious implant coatings.

Zwitterionic hydrogels (ZIHs) have also shown exceptional promise in antibiofouling applications for implantable devices due to their superhydrophilicity and charge neutrality. In a recent study, a sulfobetaine-based ZIH was covalently bonded to a polyurethane-based dural patch (NP^®^), forming an interpenetrating network (NP^®^@AZ) reinforced by hydrogen bonds [332]. This coating significantly reduced protein and bacterial adhesion, as confirmed by in vitro and in vivo tests, without relying on antibiotics. The ZIH’s lubricity also minimized post-implantation adhesion to brain tissue and bone flaps.

Advanced formulations, such as PNIPAAm–co–PMPC hydrogels, combine thermo-responsiveness with antifouling behavior, enabling temperature-triggered drug release while maintaining resistance to biofilm formation [333]. These smart hydrogels are particularly useful in wound care and implantable devices where infection risk is high. Hydrogel coatings based on polyethylene glycol (PEG) also remain a gold standard for antifouling due to their well-established safety profile and ability to minimize thrombosis and foreign body reactions [334]. However, newer materials are being developed to overcome PEG’s limitations in mechanical durability and long-term stability.

#### 6.8.2. Biointegration and Tissue Compatibility

Beyond resisting fouling, hydrogel films can be engineered to promote biointegration by facilitating cell adhesion, tissue ingrowth, and immune modulation. For example, Janus hydrogels—with asymmetric surface designs—have been developed to provide one side optimized for tissue adhesion and the other for anti-fouling and anti-wear properties [335]. These biomimetic interfaces support robust integration with soft tissues while minimizing postoperative complications.

Hydrogel-based biointerfaces also play a key role in human–machine integration, such as in neural implants and wearable electronics. Their mechanical compliance, electrical conductivity, and biocompatibility enable seamless interfacing with biological tissues, reducing foreign body responses and improving signal fidelity [9,319]. Recent studies have also explored immunomodulatory hydrogels, which can actively regulate the local immune environment to promote healing and reduce inflammation. These hydrogels are particularly promising for applications in tissue regeneration, wound healing, and implantable biosensors [336].

### 6.9. Responsive Films for Diagnostics

Hydrogel films are increasingly being developed as stimuli-responsive diagnostic platforms, offering real-time, non-invasive, and highly sensitive detection of physiological and pathological biomarkers. Their soft, hydrated, and tunable polymeric networks allow for dynamic interaction with biological environments, making them ideal for biosensing, point-of-care diagnostics, and wearable health monitoring.

#### 6.9.1. Mechanisms of Responsiveness

Responsive hydrogel films are engineered to react to external stimuli such as pH, temperature, ionic strength, light, and electric fields, or internal biological cues like enzyme activity, metabolite concentration, and biomolecular interactions [337]. These responses typically manifest as changes in swelling behavior, optical properties, electrical conductivity, or mechanical deformation, which can be transduced into measurable signals. For example, DNA-functionalized hydrogels have been used to detect progesterone, mRNA, and other biomarkers through fluorescence resonance energy transfer (FRET) and electrochemical sensing [337]. These systems offer high specificity and sensitivity, and can be tailored for multiplexed detection.

Recent advancements in biosensing technologies have increasingly focused on the detection of microRNAs (miRNAs) due to their critical role in cancer diagnostics. Among these, surface-enhanced Raman scattering (SERS) platforms have emerged as powerful tools for sensitive and multiplexed detection. Si et al. introduced a novel SERS sensor array that utilizes a dynamic “ON/OFF” Raman signal modulation strategy tailored to miRNA responsiveness (Figure 9J) [326]. This system comprises nine distinct sensing units, enabling the simultaneous identification of multiple cancer-related miRNAs within a single biological sample.

The fabrication process involved the synthesis of DNA-based hydrogels, which serve as a scaffold for the integration of AuAg nanoparticles—functioning as SERS signal tags. To enhance specificity and catalytic efficiency, multi-component nucleic acid enzymes (MNAzymes) were incorporated into the array. These enzymes facilitate target recognition and signal amplification, thereby improving the overall sensitivity of the platform. The design reflects a strategic convergence of nanomaterials and molecular biology, offering a promising route for non-invasive cancer diagnostics and real-time biomarker monitoring.

Responsive hydrogel films have been applied in various diagnostic contexts:

- Colorimetric sensors: DNAzyme-crosslinked hydrogels enable the visual detection of hydrogen peroxide (H_2_O_2_) through peroxidase-like activity, offering a simple and regenerable platform for environmental and biomedical monitoring [337].
- Electrochemical biosensors: Hydrogel films embedded with aptamers or antibodies can detect analytes such as glucose, lactate, and pathogens with high precision [337].
- Optical biosensors: Holographic hydrogel sensors diffract light in response to analyte-induced changes in refractive index, enabling label-free and real-time detection [338].
- Recent innovations include aptamer-functionalized hydrogels for continuous plasmonic biomonitoring, capable of detecting small molecules like vancomycin with high sensitivity and stability in physiological fluids [339].

#### 6.9.2. Wearable and Implantable Diagnostics

Hydrogel films are also being integrated into wearable biosensors for monitoring sweat composition, temperature, and strain. These devices leverage the mechanical compliance and biocompatibility of hydrogels to interface seamlessly with skin and tissues [340]. For instance, electrically responsive hydrogel biosensor arrays have been developed for non-invasive vascular mapping, outperforming traditional imaging techniques in locating perforating arteries [341].

The future of responsive hydrogel films in diagnostics lies in the development of multifunctional, adaptive systems that combine:

- Real-time sensing and feedback;
- Wireless data transmission;
- Integration with therapeutic platforms;
- AI-guided signal interpretation.

Emerging technologies such as 4D bioprinting, molecular imprinting, and bioelectronic interfaces are expected to further enhance the diagnostic capabilities of hydrogel films, bringing them closer to clinical translation and personalized medicine.

## 7. Recent Advances in Hydrogel Films for Biomedical Applications

Hydrogel films have evolved from simple water-retaining matrices into sophisticated, multifunctional platforms capable of responding to environmental stimuli, integrating nanomaterials, and interfacing with electronics. These innovations have significantly expanded their utility across precision medicine, diagnostics, regenerative therapies, and bioelectronics.

### 7.1. Stimuli-Responsive Hydrogel Films

Stimuli-responsive hydrogel films are engineered to undergo physical or chemical changes in response to specific triggers such as pH, temperature, light, enzymes, or reactive oxygen species (ROS). These smart materials enable on-demand drug release, dynamic tissue interaction, and real-time diagnostics, making them highly valuable in precision medicine, biosensing, and targeted therapy.

- Enzyme-responsive systems have shown promise in site-specific drug delivery and diagnostic imaging. For example, hydrogels composed of chitosan, hyaluronic acid, PEGDA, and GelMA degrade selectively in the presence of MMP-2 and hyaluronidase, releasing doxorubicin at tumor sites while sparing healthy cells. These systems also incorporate fluorescent dyes and superparamagnetic iron oxide nanoparticles (SPIONs) for dual optical and MRI-based diagnostics, demonstrating their theranostic potential [342].
- Multi-stimuli-responsive hydrogels react to combinations of triggers such as pH, temperature, light, and magnetic fields, offering precise control over therapeutic actions. These systems are being applied in cancer therapy, wound healing, and biosensing [343,344]. For instance, hydrogels that respond to acidic pH and elevated temperatures—common features of tumor microenvironments—can release chemotherapeutics only at diseased sites, reducing systemic toxicity [343]. Similarly, light-responsive hydrogels allow for spatiotemporal control of drug release or activation of therapeutic agents using external light sources [220].
- In diagnostics, these hydrogels convert environmental changes into optical, electrochemical, or mechanical signals. They can detect biomarkers such as glucose, lactate, or inflammatory enzymes, and are being integrated into wearable devices and implantable sensors [345].

### 7.2. Nanocomposite and Hybrid Hydrogel Films

Nanocomposite and hybrid hydrogel films represent a significant leap forward in the design of multifunctional biomaterials. By integrating nanostructures and polymeric diversity, these systems overcome limitations of conventional hydrogels—such as poor mechanical strength and limited bioactivity—while enabling targeted therapy, tissue regeneration, and biosensing.

Nanocomposite hydrogels incorporate nanoparticles—including carbon nanotubes (CNTs), graphene, metal oxides, and polymeric nanostructures—into hydrogel matrices to enhance mechanical strength, electrical conductivity, and biological functionality. These materials exhibit sustained therapeutic activity, reduced dosing frequency, and improved cellular interactions [53]. For example, PAM/CNT nanocomposite hydrogel films have demonstrated excellent biocompatibility, structural stability, and sustained doxorubicin release at acidic pH, effectively inhibiting breast cancer cell proliferation [43].

Hybrid hydrogels combine natural and synthetic polymers or integrate nano/microstructures to create materials with enhanced mechanical properties, controlled degradation, and tailored drug release profiles. Techniques such as click chemistry, 3D printing, and photopatterning enable precise control over structure and function [26,346].

Applications include cancer therapy, wound healing, tissue engineering, and biosensing [329], with future directions pointing toward electrically conductive hydrogels for neural interfaces, 4D-printed hydrogels, and AI-guided design [347].

### 7.3. 3D and 4D Printing of Hydrogel Films

Advances in 3D bioprinting and emerging 4D printing technologies have revolutionized hydrogel film fabrication. These printed hydrogel films are now used in drug delivery systems, tissue scaffolds, implantable devices, and soft robotics. 3D bioprinting enables the layer-by-layer deposition of hydrogel materials with spatial control over composition, porosity, and mechanical properties. Hydrogels based on GelMA, alginate, and PEG derivatives are commonly used due to their biocompatibility and crosslinking versatility. Applications include cartilage repair, bone regeneration, and vascular tissue engineering [348].

4D printing introduces time-dependent responsiveness, allowing hydrogel films to change shape or function in response to stimuli. These systems are being explored for drug delivery capsules, responsive wound dressings, and bioactuators [348].

Fabrication techniques include extrusion-based bioprinting, inkjet and laser-assisted printing, photopolymerization, and click chemistry-based modular assembly.

### 7.4. Biofunctionalization and Smart Materials

Biofunctionalized hydrogel films actively interact with biological systems through embedded bioactive molecules, peptides, or responsive moieties. These intelligent hydrogels respond to ROS, glucose, temperature, pH, and other stimuli, enabling site-specific drug delivery and real-time diagnostics [348]. Fabrication strategies such as surface functionalization, click-based orthogonal chemistry, and self-assembly allow for the fine-tuning of hydrogel behavior. Applications include artificial skin, smart drug delivery systems, and implantable medical devices.

Future directions include integration with bioelectronics, AI-guided hydrogel design, and engineered living materials (ELMs) for autonomous regeneration.

### 7.5. Integration with Wearable and Flexible Electronics

Hydrogel films are increasingly integrated into wearable and flexible electronic systems for health monitoring, diagnostics, and human–machine interfaces. Their softness, stretchability, and biocompatibility make them ideal for skin-like sensors and implantable devices. Hydrogels maintain ionic conductivity and mechanical compliance, enabling the detection of physiological signals such as strain, temperature, and biochemical markers. Transparent hydrogel-based electronics support real-time biosensing and interactive displays [349]. Advanced formulations include PEDOT:PSS/PVA organohydrogels for strain sensing and alginate–gelatin hydrogels for multimodal sensing. Applications span sweat sensors, neural interfaces, cardiac diagnostics, and interactive prosthetics [350,351,352]. Future innovations include self-healing hydrogel electronics, wireless systems using energy-harvesting hydrogels, AI-integrated biosensors, and 3D-printed hydrogel circuits.

### 7.6. Marketed Hydrogel Films Technologies and Clinical Translation

Hydrogel films have become integral to modern biomedical product development, offering a unique combination of biocompatibility, moisture retention, and mechanical flexibility. These thin polymeric matrices are engineered to interact with biological tissues, making them suitable for a wide range of clinical applications, including wound healing, drug delivery, ophthalmology, and surgical interventions [353,354].

The commercialization of hydrogel-based products has accelerated in recent years, driven by advances in polymer chemistry, fabrication techniques, and clinical demand. Products such as Intrasite Gel, Jelmyto^®^, SpaceOAR^®^, and Acuvue^®^ Oasys have received regulatory approval from agencies like the FDA and EMA, demonstrating the translational success of hydrogel technologies [355,356].

However, the path to market is governed by complex regulatory frameworks. Hydrogel films may be classified as medical devices, drug-device combination products, or advanced therapy medicinal products depending on their intended use and mechanism of action. Regulatory approval requires compliance with standards such as ISO 10993-1:2018; [357] for biocompatibility, ASTM F2900-25; [358] for hydrogel characterization, and region-specific documentation under the FDA 510(k) [359] or EU MDR 2017/745 [360] pathways.

The following tables present a curated overview of marketed hydrogel film formulations and recent active clinical trials (Table 3 and Table 4) [361]. This synthesis highlights the diversity of hydrogel technologies currently in use or under investigation, offering insight into their clinical relevance, regulatory status, and future potential.

## 8. Challenges and Limitations of Hydrogel Films for Biomedical Applications

Hydrogel films represent a frontier in biomedical innovation, offering unique advantages in biocompatibility, drug delivery, and tissue integration. Despite their promise, their clinical and commercial translation is constrained by a series of interrelated challenges—mechanical fragility, sterilization sensitivity, regulatory complexity, and economic scalability. These limitations are not isolated; they interact and compound, requiring coordinated advances in material science, process engineering, and regulatory strategy.

### 8.1. Mechanical Durability and Tear Resistance

The soft, hydrated nature of hydrogel films, while beneficial for biological compatibility, compromises their mechanical integrity. Under physiological stress, many hydrogels exhibit low fracture energy and poor tear resistance, limiting their use in load-bearing or dynamic environments. Conventional hydrogels often fall short of the mechanical benchmarks set by native tissues [362].

To overcome this, researchers have explored:

- Double-network hydrogels: Combining rigid and flexible polymer networks to enhance toughness.
- Fiber-reinforced composites: Embedding structural fibers to improve tear resistance.
- Supramolecular crosslinking: Introducing reversible bonds for elasticity and self-healing.

Some tendon-mimetic hydrogels have achieved fracture energies up to 30 kJ/m^2^ [363], approaching biological tissue performance. However, translating these innovations into thin-film formats without sacrificing flexibility, transparency, or responsiveness remains a technical bottleneck [364].

### 8.2. Sterilization and Storage Stability

Sterilization is a non-negotiable requirement for biomedical deployment; however, hydrogel films are highly sensitive to conventional methods:

- Autoclaving and gamma irradiation can disrupt polymer networks, degrade bioactive agents, and alter swelling behavior.
- Ethylene oxide treatment, while gentler, introduces residual toxicity concerns.

Emerging alternatives like supercritical CO_2_ and cold plasma sterilization offer promise but require specialized infrastructure and regulatory validation [365].

Storage stability poses additional hurdles. Hydrogel films are prone to:

- Water loss, leading to shrinkage and loss of functionality.
- Microbial contamination, especially in bioactive formulations.
- Chemical degradation, stimuli-responsive behavior, and drug release profiles [366,367].

Maintaining sterility and performance over extended shelf lives, especially for smart or biofunctionalized hydrogels, remains a critical challenge [368].

### 8.3. Regulatory Requirements and Clinical Translation

Hydrogel films offer transformative potential in biomedical applications, including wound healing, drug delivery, and tissue engineering. However, their clinical translation is governed by complex and jurisdiction-specific regulatory frameworks. Navigating these frameworks is not merely procedural—it is strategic, influencing product design, development timelines, and market access.

#### 8.3.1. Classification and Regulatory Pathways

Hydrogel films may be regulated as medical devices, drug-device combination products, or advanced therapy medicinal products (ATMPs), depending on their intended use and mechanism of action.

- FDA (USA): Under the FD&C Act, hydrogel films are categorized into Class I, II, or III devices based on risk. Most wound dressings fall under Class II and require a 510(k) premarket notification. Drug-loaded hydrogels may require Premarket Approval (PMA) or be treated as combination products, involving both CDRH and CDER oversight [369].
- EMA (EU): The MDR (EU 2017/745) [370] imposes stricter requirements, including clinical evaluation and post-market surveillance. Hydrogels with pharmacological activity are classified as combination products and require dual compliance with MDR and Directive 2001/83/EC [371].

Understanding these classifications is essential for selecting the appropriate regulatory pathway and aligning product development with approval requirements.

#### 8.3.2. Biocompatibility and Safety Testing

Safety validation is a cornerstone of regulatory approval. Hydrogel films must undergo comprehensive biocompatibility testing under ISO 10993-1:2018 standards [357]:

- ISO 10993-5:2009 [372]: Cytotoxicity;
- ISO 10993-10:2010 [373]: Irritation and sensitization;
- ISO 10993-11:2017 [374]: Systemic toxicity.

Additionally, ASTM F2900-25;2025 [358] provides hydrogel-specific guidance on swelling behavior, degradation kinetics, and agent release profiles. These tests must be conducted under Good Laboratory Practices (GLP) and are prerequisites for both CE marking and FDA approval.

#### 8.3.3. Documentation and Submission Strategy

Regulatory submissions require extensive documentation to demonstrate safety, efficacy, and manufacturing control:

- Technical File (EU) or Device Master File (USA);
- Material characterization and source traceability;
- Sterilization validation (e.g., gamma, ethylene oxide, aseptic processing);
- Shelf-life and packaging stability;
- Clinical performance data (in vitro/in vivo).

Early engagement with regulators is recommended. The FDA’s Q-submission program facilitates pre-submission dialogue, while the EMA mandates interaction with a Notified Body for conformity assessment [375,376].

#### 8.3.4. Challenges in Regulatory Approval

Several intrinsic challenges complicate the approval of hydrogel films:

- Sterilization Sensitivity: Conventional methods (e.g., gamma irradiation) may degrade hydrogel structure, necessitating alternatives like low-temperature plasma or aseptic manufacturing [377].
- Batch-to-Batch Variability: Natural hydrogels (e.g., alginate, chitosan) exhibit variability in source material, affecting reproducibility.
- Combination Product Complexity: Drug-loaded hydrogels require coordination between multiple regulatory divisions, increasing approval complexity [377].

These challenges often delay clinical translation and increase development costs.

#### 8.3.5. Standards and Harmonization

Beyond ISO 10993 [357], several ASTM standards [358] support reproducible testing and regulatory alignment:

- ASTM F748-25 [378]: Biological test method selection.
- ASTM F2027-16 [379]: Raw biomaterial characterization.
- ASTM F2064-17 [380]: Testing of alginates.
- ASTM F2103-18 [381]: Testing of chitosan salts.
- ASTM F2900-11 [382]: Hydrogel-specific testing in regenerative medicine.

These standards provide a harmonized framework for regulatory submissions and are frequently referenced by both the FDA and EMA.

#### 8.3.6. Strategic Implications for Clinical Translation

Despite their promise, hydrogel films face significant regulatory hurdles. Globally, fewer than 100 hydrogel-based products have received clinical approval, reflecting the difficulty of navigating regulatory pathways [383]. Long approval timelines (7–12 years), high development costs, and a lack of commercialization support often confine promising technologies to academic labs. Strategic collaboration between academia, industry, and regulatory bodies is essential to accelerate clinical translation and unlock the full potential of hydrogel films.

### 8.4. Cost and Scalability of Production

Scaling hydrogel film production from lab to industry presents significant technical and economic challenges. Traditional batch processes struggle with consistency, while mixing, casting, and drying operations require precise control to maintain product quality [384].

Drying methods such as freeze-drying and supercritical drying are effective but energy-intensive and expensive, limiting feasibility for large-scale manufacturing [385]. In-line monitoring technologies for real-time quality control are underdeveloped, increasing waste and production costs.

The average production cost of hydrogel films remains 30–40% higher than traditional alternatives, creating barriers in price-sensitive markets [386]. Innovations in automation, modular manufacturing, and sustainable formulations are needed to reduce costs and improve scalability.

### 8.5. Integrated Outlook and Strategic Recommendations

The limitations of hydrogel films—mechanical fragility, sterilization sensitivity, regulatory complexity, and production costs—are deeply interconnected. Mechanical weakness affects sterilization tolerance; sterilization impacts regulatory approval; and regulatory hurdles increase development costs. Overcoming these barriers demands multidisciplinary innovation, strategic collaboration, and a systems-level approach to design, testing, and commercialization.

Only through coordinated efforts between academia, industry, and regulatory bodies can the full potential of hydrogel films be realized in clinical practice.

## 9. Critical Discussion and Future Directions

### 9.1. Critical Gaps, Conflicting Evidence, and the Imperative for Standardization

While hydrogel films have demonstrated significant promise across biomedical domains, a critical comparative analysis revealed several persistent challenges and knowledge gaps that warrant further investigation. For instance, although natural biopolymer-based hydrogels (e.g., alginate, chitosan, gelatin) offer superior biocompatibility and biodegradability, they often suffer from poor mechanical strength and batch-to-batch variability, limiting their scalability and long-term stability [112]. In contrast, synthetic hydrogels (e.g., PVA, PEG, PNIPAM) provide tunable mechanical properties and reproducibility [114,115], but may lack intrinsic bioactivity and pose risks of cytotoxicity due to residual monomers or crosslinkers [113]. Composite hydrogels attempt to bridge this divide, but the optimal balance between mechanical robustness and biological functionality remains elusive [113].

Conflicting results are also evident in the literature regarding the in vivo degradation rates and immune responses elicited by different hydrogel formulations. For example, while some studies report minimal inflammatory response with PEG-based hydrogels [113], others highlight the potential accumulation of toxic degradation byproducts, especially in long-term applications [325]. Similarly, the efficacy of stimuli-responsive hydrogels in clinical settings remains underexplored, with most data derived from in vitro or small-animal models, raising concerns about translational relevance [111].

Moreover, despite the proliferation of fabrication techniques—ranging from solvent casting to 3D printing—there is a lack of standardized protocols for evaluating film uniformity, crosslinking efficiency, and functional performance [112]. This heterogeneity hampers direct comparison across studies and complicates regulatory approval pathways [113]. Additionally, sterilization methods such as gamma irradiation or ethylene oxide often compromise hydrogel integrity, but alternative techniques like supercritical CO_2_ or cold plasma remain underutilized and insufficiently validated [325].

### 9.2. Future Directions and Strategic Perspectives for Hydrogel Film Technologies

Hydrogel films are poised to play a transformative role in next-generation biomedical technologies. As research advances, the focus is shifting toward personalized, intelligent, sustainable, and clinically translatable hydrogel systems. This section explores emerging directions that are expected to shape the future of hydrogel film development and application.

#### 9.2.1. Personalized and Patient-Specific Hydrogel Films

The integration of hydrogel films into precision medicine is gaining momentum. Personalized hydrogel systems are being designed to match individual patient needs, including tissue-specific mechanical properties, drug release profiles, and biological compatibility. Recent developments in 3D bioprinting have enabled the fabrication of patient-specific hydrogel constructs for organ models, wound dressings, and tissue scaffolds [173]. These systems can be tailored using patient-derived cells and biomaterials, allowing for customized therapeutic interventions. Moreover, biomaterial-based hydrogels are being explored for personalized drug delivery, where the hydrogel matrix is optimized for the patient’s metabolic profile and disease state [387]. This approach enhances therapeutic efficacy and minimizes adverse effects.

#### 9.2.2. AI-Guided Design and Optimization

Artificial Intelligence (AI) and Machine Learning (ML) are revolutionizing hydrogel design by enabling predictive modeling, property optimization, and automated formulation discovery. AI-driven platforms can analyze complex datasets to identify optimal polymer compositions, crosslinking strategies, and drug release kinetics [388]. For example, Bayesian optimization and neural networks have been used to fine-tune polyacrylamide/alginate hydrogels for flexible electronics and biomedical sensors [389]. These computational tools accelerate development timelines, reduce experimental costs, and facilitate the creation of multi-functional hydrogel films with enhanced performance [390].

#### 9.2.3. Sustainable and Biodegradable Materials

Sustainability is becoming a core criterion in hydrogel film development. Researchers are increasingly turning to bio-based and biodegradable polymers such as bacterial cellulose, recombinant collagen, silk fibroin, and xanthan gum [391]. These materials are produced via biotechnological methods like microbial fermentation and genetic engineering, reducing reliance on petrochemical sources and minimizing environmental impact. Sustainable hydrogels are being used in wound healing, tissue regeneration, and drug delivery, aligning with the One Health paradigm that integrates human, animal, and environmental health [392]. Natural polymer-based hydrogels also offer biodegradability, reducing long-term waste and improving safety in implantable applications [393].

#### 9.2.4. Clinical Trials and Commercialization Pathways

Hydrogel films are transitioning from laboratory research to clinical and commercial use. Several hydrogel-based products have received FDA and EMA approval, while others are undergoing active clinical trials for applications in drug delivery, wound care, and tissue engineering [6]. Challenges in clinical translation include regulatory classification (device vs. drug), long approval timelines (7–12 years), and manufacturing scalability. However, recent reviews highlight the successful commercialization of hydrogel products for ocular, transdermal, and injectable applications [393]. The global hydrogel market is projected to grow significantly, driven by innovations in stimuli-responsive systems, wearable diagnostics, and bioprinted implants. Strategic partnerships between academia, industry, and regulatory bodies are essential to accelerate commercialization and ensure safety and efficacy [370].

The future of hydrogel films lies in their ability to adapt to individual patient needs, integrate intelligent design frameworks, and align with sustainability goals. Advances in AI, bioprinting, and biomaterials science are converging to create hydrogel systems that are not only functional but also ethical and environmentally responsible. As clinical trials expand and commercialization pathways mature, hydrogel films are expected to become central to personalized and regenerative healthcare.

## 10. Conclusions

This review has comprehensively examined the structural diversity, fabrication strategies, and biomedical applications of hydrogel films. These materials, derived from natural, synthetic, and composite polymers, exhibit tunable mechanical, chemical, and biological properties that make them highly adaptable for applications in wound healing, drug delivery, tissue engineering, ophthalmology, and biosensing. Advances in fabrication techniques—such as photopolymerization, microfluidics, and 3D/4D printing—have enabled the development of hydrogel films with enhanced responsiveness, multifunctionality, and integration with electronic systems.

Despite these advancements, several critical challenges remain. Mechanical fragility, limited long-term stability, and inconsistent degradation profiles hinder clinical reliability. The lack of standardized evaluation protocols and the complexity of regulatory classification (e.g., device vs. combination product) further complicated clinical translation. Moreover, scalable manufacturing remains a significant bottleneck, as many current fabrication methods are not yet optimized for industrial throughput or cost-efficiency. Environmental sustainability is another emerging concern, particularly regarding the use of non-biodegradable synthetic polymers and toxic crosslinkers.

To address these gaps, future research should focus on three strategic priorities: (1) developing robust, scalable, and GMP-compliant manufacturing platforms; (2) aligning material design and testing with evolving regulatory frameworks; and (3) advancing the use of environmentally sustainable, biodegradable, and bio-based polymers. Additionally, integrating AI-driven design, green crosslinking chemistries, and modular fabrication strategies may accelerate the clinical and commercial readiness of hydrogel films.

By addressing these limitations through interdisciplinary collaboration among materials scientists, biomedical engineers, clinicians, and regulatory bodies, hydrogel films can transition from promising laboratory constructs to transformative clinical solutions in personalized and regenerative medicine.

## Figures and Tables

**Table 1:** Comparative Advantages and Disadvantages of Hydrogel Film Types.

Hydrogel Type	Advantages	Disadvantages	Refs.
Natural Biopolymer-Based Hydrogels	- Biocompatible and biodegradable - Mimics extracellular matrix - High water absorption - Sustainable and low-cost - Suitable for wound healing and drug delivery	- Poor mechanical strength - Batch-to-batch variability - Limited scalability - Sensitive to environmental conditions	[64,65,66,68,70,71,72,79,81,83,84,85,86,87,151]
Synthetic Polymer-Based Hydrogels	- Tunable mechanical and chemical properties - Scalable and reproducible - Stimuli-responsive (pH, temperature, light) - High structural integrity - Industrially viable	- Potential toxicity from residual monomers or crosslinkers - Requires purification for biomedical use - May lack bioactivity without modification	[112,113,116,117,118,120,121,124,129,152]
Additive-Enhanced Composite Hydrogels	- Enhanced mechanical, thermal, and barrier properties - Functional versatility (e.g., antibacterial, magnetic, conductive) - Enables targeted drug delivery and sensing - Synergistic effects from multiple additives	- Complex synthesis and dispersion challenges - Potential cytotoxicity of some nanoparticles - Cost and scalability issues for advanced additives	[132,133,135,136,137,138,140,146,148]

**Table 2:** Comparison of Film Fabrication Techniques [166,176,177].

Technique	Cost	Scalability	Uniformity	Min. Thickness	Patterning In Situ	Advantages	Disadvantages	Limitations
Solvent Casting	Low	Limited	Moderate	Tens of µm	Yes	Simple, low-cost, compatible with many polymers	Residual solvent, weak mechanical strength	Poor thickness control, limited scalability
Dip Coating	Low	Low	High	Nanometers	Yes	Uniform coating on complex shapes	Slow drying, limited scalability	Not suitable for large-area films
Spin Coating	Medium	Moderate	High	Nanometers	No	Excellent uniformity, thin films	Restricted to small, flat substrates	Requires precise control, limited scalability
Spray Coating	High	Moderate	Low	Tens–hundreds nm	No	Fast, adaptable to various geometries	Low uniformity, high equipment cost	Difficult thickness control
Blade Coating	Medium	Limited	Moderate	Tens of µm	Yes	Good thickness control, scalable	Less uniformity, flat surfaces only	Not ideal for ultra-thin films
Bar Coating	Medium	High	Moderate	Nanometers	No	Simple setup, scalable	Limited precision, slower process	Cannot create gradient or patterned films
Slot Die Coating	High	High	High	Tens–hundreds nm	Limited	Precise thickness control, industrial scalability	Complex setup, expensive	Requires optimization for each material
Photolithography	Very High	Low	Very High	Micrometers	Yes	High-resolution patterning	Extremely costly, complex process	Limited to specific substrates
3D Printing	High	Low	Moderate	Micrometers	Yes	Custom geometries, material-efficient	Slow, limited scalability	Best for prototyping, not mass production
Electrospinning	Medium	High	High	Nanometers	Limited	Produces nanofibers with, high surface area	Requires high voltage, limited patterning	Complex setup, material restrictions

**Table 3:** Commercially Available Hydrogel Film Products for Biomedical Applications.

Patent No./Country	Title	Application	Indication	Composition	Key Features
US10799696B2/United States	Polymer formulations for nasolacrimal stimulation	Ophthalmic Device	Dry eye	UV-crosslinked hydrogel	Permits electrical stimulation of the lacrimal gland to increase tear production
US20200085733A1/United States	Hypotonic hydrogel formulations for enhanced transport of active agents at mucosal surfaces	Drug Delivery System	Vaginal or colorectal administration	Aqueous polymeric hydrogel (poloxamers)	Forms a barrier plug and delivers agents to mucosal surfaces
US20200114010A1/United States	Non-injectable hydrogel formulations for smart release	Drug Delivery System	Oral, rectal, vaginal administration	Self-assembling gelators	Formulated as capsules, tablets, suppositories, etc.
US20180023049A1/United States	Synthetic peptide hydrogel formulations for use as an extracellular matrix	Tissue Engineering Scaffold	Cell culture experimentation	Synthetic peptide hydrogel	pH ~3.5, isotonic osmolality range
US20200360281A1/United States	A thermo-responsive hydrogel for intertumoral administration as a treatment for solid tumor cancers	Drug Delivery System	Solid tumor cancer	Chitosan and genipin interpenetrating scaffold	Injectable, retains thermo-responsiveness
US20190127726A1/United States	Delivering an enzyme using an injectable hydrogel depot	Drug Delivery System	Enzyme delivery	Injectable enzyme hydrogel	Depot formulation for sustained enzyme release
WO2017152112A2/WIPO (PCT)	Hydrogel systems for skeletal interfacial tissue regeneration applied to epiphyseal growth plate repair	Tissue Engineering Scaffold	Epiphyseal growth plate repair	Biomaterials and hydrogel systems	Guides the regeneration of interfacial tissue structures
CN105209016B/China	Biocompatible hydrogel polymer matrices for cell delivery	Tissue Engineering Scaffold	Cell delivery	Bioabsorbable hydrogel polymer matrices	Controlled release at the target site
JP2020514500A/Japan	Antibacterial polymer and antibacterial hydrogel	Wound Dressing	Antimicrobial treatment	Polyethyleneimine-alkyl-polyethylene glycol methacrylate	Antibacterial hydrogel coating for devices
EP2801377B1/European Patent Office	Hydrogel comprising cells for local release of growth factors to mediate motor recovery after stroke	Tissue Engineering Scaffold	Stroke recovery	Hydrogel with embedded cells	Sustained release of BDNF
JP6293254B2/Japan	Silicone hydrogel lens with crosslinked hydrophilic coating	Ophthalmic Device	Contact lens	Silicone hydrogel with crosslinked hydrophilic coating	Improved comfort and wettability
JP6066237B2/Japan	Antibacterial ophthalmic contact lenses	Ophthalmic Device	Antibacterial contact lens	Hydrogel with epsilon polylysine	Non-covalent bonding of εPLL for antimicrobial effect
EP3151872B1/European Patent Office	Wound dressing	Wound Dressing	Moist wound healing	Lyophilized hyaluronic acid hydrogel with chitosan and hypromellose	Stimuli-responsive, promotes angiogenesis
WO2019221559A1/WIPO (PCT)	Microneedle adhesive patch based on hydrogel formulation	Drug Delivery System	Wound regeneration	Hydrogel layers with mussel adhesive protein, hyaluronic acid, and silk fibroin	Biocompatible, biodegradable, strong tissue adhesion
WO2020036526A1/WIPO (PCT)	A biphasic hydrogel formulation and methods of production and use thereof	Drug Delivery System	Skin conditions (e.g., sunburn, acne)	Biphasic hydrogel with liquid and elastic layers	Cooling effect and immediate drug release
US20200246472A1/United States	Hydrogel-forming composition for controlled release	Drug Delivery System	Controlled drug release	Peptide hydrogelators	Injectable biogel for sustained release
US10471181B2/United States	Fiber-hydrogel composite surgical meshes for tissue repair	Tissue Engineering Scaffold	Surgical mesh integration	Hydrogel/nanofiber composite	Reduces foreign body response, improves integration

**Table 4:** Active Clinical Trials Investigating Hydrogel-Based Biomedical Formulations.

Hydrogel Type	Medical Condition	Delivery Method	Clinical Findings	Trial Status	Trial ID
Hydroxyethyl cellulose hydrogel	Osteoarthritis-related knee pain	Injection	Not available	Ongoing	NCT04061733
Polyacrylamide hydrogel	Osteoarthritis-related knee pain	Intra-articular injection	Clinical improvement from −7 to 7 on the scale	Completed	NCT03060421
Polyacrylamide + Hyaluronic acid hydrogel	Osteoarthritis-related knee pain	Intra-articular injection	Not available	Ongoing	NCT02763956
Polyacrylonitrile hydrogel	Degenerative disc disease	Intra-discal injection	Not available	Ongoing	NCT02763956
Alginate hydrogel	Heart failure	Intra-myocardial injection	Enhanced oxygen uptake	Completed	NCT01311791
Renal cells gelatin hydrogel	Kidney disease	Injection	Improved creatinine, proteinuria, and GFR	Completed	NCT02525263
Renal cells gelatin hydrogel	Congenital chronic kidney disease	Injection	Not available	Ongoing	NCT04115345
Human amniotic epithelial cells hydrogel	Asherman’s syndrome	Intra-uterine injection	Not available	Ongoing	NCT03223454
Cardiac stem cells gelatin hydrogel	Ischemic cardiomyopathy	Intra-myocardial injection	Improved ventricular function	Completed	NCT00981006
Radiopaque hydrogel	Pancreatic cancer	Injection	Not available	Ongoing	NCT03307564
Biosentry hydrogel	Pneumothorax risk post-lung biopsy	Tract plug	Not available	Ongoing	NCT02224924
TraceIT hydrogel	Oropharyngeal cancer	Injection	Not available	Ongoing	NCT03713021
TraceIT hydrogel	Rectal cancer	Transperineal injection	Not available	Ongoing	NCT03258541
SpaceOAR hydrogel (PEG)	Radiation-induced rectal damage prevention	Injection	Reduced adverse effects and radiation exposure	Completed	NCT01538628
SpaceOAR hydrogel	Prostate cancer radiotherapy	Injection	Reduced rectal toxicity	Completed	NCT02212548
TraceIT hydrogel	Bladder cancer radiotherapy	Injection	Improved tumor localization during imaging	Completed	NCT03125226
VentriGel	Myocardial infarction and heart failure	Trans-endocardial injection	Improved EF, EDV, and ESV	Completed	NCT02305602
Gut Guarding Gel (alginate + calcium lactate)	Post-endoscopic submucosal dissection	Sub-mucosal injection	Enhanced mucosa formation and reduced bleeding	Completed	NCT03321396
Polyacrylamide hydrogel	Urinary incontinence	Transurethral injection	Improved bladder retention and voiding	Completed	NCT02776423
Polyacrylamide hydrogel + Botox	Urinary incontinence	Midurethral injection	Increased micturitions and symptom relief	Completed	NCT02815046
Polyacrylamide hydrogel	Anal incontinence	Transanal injection	Reduced Wexner scores	Completed	NCT02550899
OTX-TKI (PEG hydrogel + tyrosine kinase inhibitor)	Age-related macular degeneration	Intravitreal injection	Not available	Ongoing	NCT03630315

## Data Availability

No new data were created or analyzed in this study.
